# Comprehensive comparison of potential flavor-active peptides, amino acids and pigments accumulation in different altitudes cultivated albino teas

**DOI:** 10.1016/j.fochx.2025.102722

**Published:** 2025-07-03

**Authors:** Yan Kangni, Yang Jiaqi, Zhou Mengxue, Peng Qunhua, A. Bassiony, Bai Xue, Feng Shan, Wang Jiatong, Lin Zhi, Mu Dan, Fu Jianyu, Wu Yan, Lv Haipeng, Shi Jiang

**Affiliations:** a School of Life Sciences, Key Laboratory of Biodiversity Conservation and Characteristic Resource Utilization in Southwest Anhui, Anqing Forestry Technology Innovation Research Institute, Anqing Normal University, Anqing 246133, Anhui, China; b Key Laboratory of Tea Biology and Resource Utilization of Ministry of Agriculture, Tea Research Institute, Chinese Academy of Agricultural Sciences, Hangzhou 310008, China; c Mass Spectrometry & Metabolomics Core Facility, The Biomedical Research Core Facility, Westlake University, Hangzhou 310024, China

**Keywords:** Albino tea, Umami peptide, Bitter peptides, Flavor peptide-taste protein complexes, Pigments

## Abstract

Albino tea was known for umami and exhibited varying flavors responding to cultivation and processing, necessitating further exploration. In this study, peptidomics and targeted metabolomics unraveled crucial taste related compounds in low-altitude (L) and high-altitude (H) samples. >300 peptides were newly identified via de novo sequencing, with 24 umami and 19 bitter peptides predicted with potential active cores. Total amino acids content was higher in L (31.62 mg/g) than in H (28.35 mg/g), with theanine and glutamic acid accounting for 54.29 % and 2.02 %, compared to 54.95 % and 7.47 % in H, respectively. Lutein and β-carotene were more abundant at L (> 400 μg/g) than at H (∼200 μg/g), and significantly increased during processing. Epigallocatechin gallate reached 65.18 mg/g in L that higher than H (58.66 mg/g). These findings expanded the understanding of flavor peptides in albino tea and will underscore the importance of altitude-specific strategies achieving premium quality.

## Highlights

• 326 peptides in ‘Baiye 1’ tea leaves newly identified by de novo sequencing
• 78 peptides were unique to L_FLs, 193 peptides were unique to H_FLs
• 24 potential umami and 19 bitter flavor peptides were predicted by modeling
• Low-altitude leaves had more amino acids, especially theanine and glutamic acid
• Carotenoids like lutein and β-carotene were more abundant in low-altitude tea

## 1 Introduction

Tea (*Camellia sinensis*) is a globally consumed non-alcoholic beverage appreciated for its diverse flavors and potential health benefits (Hinojosa-Nogueira et al., 2021). Among the various tea types, albino tea stands out due to its pale leaf colour and distinct umami taste. This unique appearance results from a reduction in chlorophyll content, which alters the leaf's biochemical pathways and, in turn, affects the sensory profile of the tea (Li et al., 2011; Zhao et al., 2024). The flavor of albino tea is primarily shaped by amino acids (AAs), flavonoids, and other primary metabolites like alkaloids, nucleotides, peptides, and sugars. These components collectively contribute to the tea's balance of umami, bitterness, and sweetness (Fu et al., 2021; Zhao et al., 2023). Albino cultivars, especially ‘Baiye 1’ have been shown to accumulate higher concentrations of *L*-theanine and *L*-glutamic acid, key contributors to the umami flavor (Zhu et al., 2023; Chen, Y., Han, Y., & Tong, H, 2024). Flavonoids, another major class of compounds, are primarily responsible for the bitterness and astringency of tea. In albino teas, reduced chlorophyll leads to lower biosynthesis of catechins, resulting in milder bitterness compared to conventional green teas (Ye et al., 2022). Recent research has highlighted the role of oligopeptides in enhancing the complexity of flavor, particularly in modulating umami and bitterness in mushroom and seafood (Yuan et al., 2022; Zhang et al., 2022; Zhang, Yan, Peng, Baldermann, et al., 2024). While peptides compositions have been well studied in common green teas, their potential flavor contributions remain largely unexplored (Kumar et al., 2023; Zhang et al., 2021; Zhao et al., 2022).

Tea flavor and quality are influenced by numerous factors, including tea cultivar, growing environment, and post-harvest processing. Among these, altitude represents a critical ecological factor that integrates variables such as temperature, humidity, light exposure, and soil properties. These factors affect the synthesis of secondary metabolites and consequently impact flavor compound accumulation. Many studies have reported that higher altitudes are associated with improved tea quality, typically due to enhanced accumulation of amino acids—imparting a fresh and sweet taste—while lower altitudes often promote the biosynthesis of catechins and caffeine, leading to more bitter and astringent profiles (Bassiony et al., 2024). For instance, Chen et al. (2010) observed significantly higher EGCG, CG, and total catechin contents in high-altitude oolong teas compared to low-altitude counterparts. Similarly, Wang et al. (2021) and Sun et al. (2024) reported that green teas from high elevations exhibit elevated levels of amino acids, sugars, and organic acids, but reduced catechin content.

Tea is categorised into six types based on fermentation: green, white, yellow, oolong, black, and dark tea, each with unique processing methods contributing to distinctive flavor profiles. Green tea, as a non-fermented type, retains a refreshing taste. This freshness is linked to a range of complex biochemical transformations occurring during processing, including withering and drying. Ye et al. (2018) demonstrated that with extended spreading time and increased temperature, the content of most free amino acids rose while catechin levels declined. Other studies have further shown that heat treatments during green tea production significantly reduce catechin concentrations while increasing the levels of flavone-C-glycosides and lipid compounds (Yu & Yang, 2020).

Despite these insights, limited systematic research exists regarding how different altitudes and green tea processing affect the flavor and chemical profiles of the ‘Baiye 1’ albino cultivar. Therefore, the objective of this study was to comprehensively evaluate the impact of altitude on the accumulation of flavor-related metabolites—amino acids, flavonoids, carotenoids, and novel water-soluble peptides—in fresh leaves of ‘Baiye 1’. Using integrative metabolomic and peptidomics analyses, we also aimed to elucidate the dynamic changes in these compounds during green tea processing. The findings of this study will provide valuable scientific evidence for optimising cultivation and processing strategies aimed at enhancing the flavor quality of albino tea cultivars.

## 2 Materials and methods

### 2.1 Chemicals and reagents

Methanol, ethanol, acetone, and formic acid were all in MS grade(purity≥99 %) and purchased from Sigma Co. (St. Louis, USA). Milli-Q water (Millipore Co., Bradford, MA, USA) was used in all experiments. Flavonoid standards (purity HPLC >98 %), including epigallocatechin gallate (EGCG), epicatechin gallate (ECG), epigallocatechin (EGC), epicatechin (EC), gallocatechin (GC), catechin gallate (CG), gallocatechin gallate (GCG), catechin (C), myricetin, myricetin 3-galactoside (M-3-gal), myricetin 3′-glucoside (M-3′-glu), quercetin, quercetin 3-galactoside (Q-3-gal), quercetin 3-glucoside (Q-3-glu), kaempferol, kaempferol 3-rutinoside (K-3-rut), and myricetin were purchased from Olchemim Ltd. (Olomouc, Czech Republic) and Sigma Co. (St. Louis, USA). The flavonoids stock solutions of standards were prepared at a concentration of 5 mg/mL. Twenty amino acids (purity >98 %), Twenty AAs, including phenylalanine (Phe), leucine (Leu), tryptophan (Try), isoleucine (Ile), valine (Val), proline (Pro), tyrosine (Tyr), cysteine (Cys), alanine (Ala), threonine (Thr), glycine (Gly), glutamine (Gln), serine (Ser), glutamic acid (Glu), aspartic acid (Asp), histidine (His), asparagine (Asn), arginine (Arg), lysine (Lys), and theanine, were all purchased from Sigma Co. (St. Louis, USA). AAs standards stock was prepared at a concentration of 2 mg/mL. Carotenoids standards stock were prepared at a concentration of 1 mg/mL. All stock solutions were stored at −20 °C until used.

### 2.2 Samples preparation and characterized taste flavor sensory evaluation

‘Baiye 1’ were harvested from table land tea gardens as low altitude tea garden (∼80 m, L_FLs) in Shengzhou, Shaoxing city, Zhejiang province (120°49′E; 29°35′N), by collecting one bud with two leaves for subsequent green tea production. All fresh leaves (FLs) were collected in March 2023, and green tea products were manufacturing. Samples were collected during crucial processing steps, including fresh leaves(L_FLs) → spreading (L_SP) → killing-green→ rolling → drying (L_GT), and immediately frozen in liquid nitrogen, followed by freeze-drying and stored at −80 °C. Meanwhile, FLs from high-altitude tea gardens (∼600 m, H_FLs) in the same location underwent identical collection and processing procedures and defined as H_SP, H_GT.

Five well-trained panelists who had >10 years' experience (1 males and 4 females, aged from 23 to 40 years,) were employed to perform the sensory evaluation. The national standard “Methodology of sensory evaluation of tea” (GB/T 23776–2018) was used as the sensory evaluation method. Briefly, a 3 g tea sample was infused with 150 mL of boiled water in a white porcelain cup. After 4 min of brewing, the tea infusion was separated by filtration. All panelists were required to give description of samples share appearance, aroma and taste description and score (0−100), which represented to the lowest to highest quality. Sensory evaluations focused on the umami and mellow taste, and sweet, and bitter intensities of each sample. Sensory evaluations focused on the umami and mellow taste, and sweet, and bitter intensities of each sample. The “Ethics statement” has been included in the relevant section. General sensory evaluation results were shown in Table S1.

### 2.3 Qualification of water-soluble peptides in ‘Baiye 1’

Tea samples were ground into a fine powder. 50 mg of powder (accurate to 0.001 g) were weighed into a 1.5 mL centrifuge tube, 1 mL of 95 °C distilled water was added, and the mixture was shaken in a 95 °C water bath for 5 min. After centrifugation (10,000 *g*, 30 min, 4 °C), the supernatant was filtered through ultrafiltration tube (10 KD) and vacuum dried. The dried peptides were reconstituted in 20 μL of mobile phase A buffer. High-throughput peptide identification was performed using a 125 min gradient elution at a flow rate of 0.300 μL/min with the Thermo Vanquish integrated nano-HPLC system, which was directly interfaced with the Thermo Q Exactive HF-X mass spectrometer. The analytical column was a home-made fused silica capillary column (75 μm ID, 150 mm length; Upchurch, Oak Harbor, WA) packed with C-18 resin (300 A, 3 μm, Varian, Lexington, MA). Mobile phase A consisted of 0.1 % formic acid, and mobile phase B consisted of 80 % acetonitrile and 0.1 % formic acid. The mass spectrometer was operated in the data-dependent acquisition mode using the Xcalibur 4.1 software and there is a single full-scan mass spectrum in the Orbitrap (400–1800 *m*/*z*, 60,000 resolution) followed by 20 data-dependent MS/MS scans at 30 % normalized collision energy. Peaks Studio 11 software (Bioinformatics Solutions Inc., Waterloo, Ontario, Canada) was applied for peptide de novo sequencing; peptide mass tolerance: 10 ppm; fragment mass error tolerance: 0.01 Da; enzyme: none; ALC (average local confidence) scores ≥99 % were set as indexes.

### 2.4 Targeted metabolomic analysis of 19 essential AAs and *L*-theanine

Fifty milligram of lyophilized samples were finely ground and supplemented with 1 mL of 80 % (*v*/v) HPLC-grade methanol (pre-chilled at −80 °C) in a 2 mL Eppendorf tube, vortexed for 2 min at 4 °C, and incubated at −80 °C for 2 h. Samples were centrifuged at 14,000 *g* for 20 min at 4 °C, successively transferring the same volume of supernatant to a new Eppendorf tube. A SpeedVac with N_2_ was used to dry the pellet at room temperature. Prior to LC-MS analysis, the dried extracts were reconstituted in 500 μL 80 % methanol. Chromatographic elusion was performed using an Agilent 1290 infinity system (Agilent Technologies, Santa Clara, CA, USA) equipped with a sample manager coupled to a mass spectrometer with an electrospray ion source in both positive and negative ion modes. Separation was carried out on the BEH amide column (100 mm × 2.1 mm, 1.7 μm) at 40 °C Mobile phase A was ultrapure water containing 0.3 % ammonia and 15 mM ammonium acetate, and mobile phase B was acetonitrile/water (9:1) containing 0.3 % ammonia and 15 mM ammonium acetate. The flow rate was 0.3 mL/ min, and the gradient for mobile phase A was as follows: holding at 5 % for 1 min, 5 % to 50 % in 8 min, holding at 50 % for 3 min, 50 % to 10 % in 0.5 min, and finally holding at 5 % for 6.5 min. The sample volume injected was 2 μL. An Agilent 6545 Q/TOF-MS system in both ESI+/− modes was used. The parameter settings were as follows: capillary voltage, 3500 V; Nozzle voltage, 120 V; nebuliser gas, 35 psi; drying gas flow rate, 8 L/min; and gas temperature, 350 °C. A full scan range from *m*/*z* 50 to 1200. AAs were quantitatively analysis by standards verification and calculated using standard curves.

### 2.5 Quantitative analysis of major pigments

Adapting our previous methodologies (Zhang, Yan, Peng, Feng, et al., 2024), lyophilized tea samples were extracted and analyzed using an ultra-high performance liquid chromatography-quadrupole/electrostatic field orbitrap mass spectrometer system (UHPLC-Q-Exactive/MS, Thermo Fisher Scientific, Waltham, MA, USA). The contents of 8 flava-3-ols, and 11 flavonol glycosides were determined using the external standard method by preparation of a mixture of standard working solutions.

Quantification of major tea carotenoids was carried out strictly according to our previous method (Shi, Wu, et al., 2023), including carotenoids extraction with organic solvents, UPLC-APCI-MS/MS, and quantitative analysis.

### 2.6 Multivariate analysis

All tests were repeated in triplicate, and the results of each test are expressed as the average of three replicates. Statistical analyses were performed using SPSS software (version 26.0). Plotting was done using GraphPad Prism 9.5. HCA analyses were performed using a comprehensive toolkit, TBtools.

## 3 Results and discussions

### 3.1 Identification of water-soluble peptides in ‘Baiye 1’ fresh leaves (FLs) harvested from low and high altitudes

**Fig. 1:**
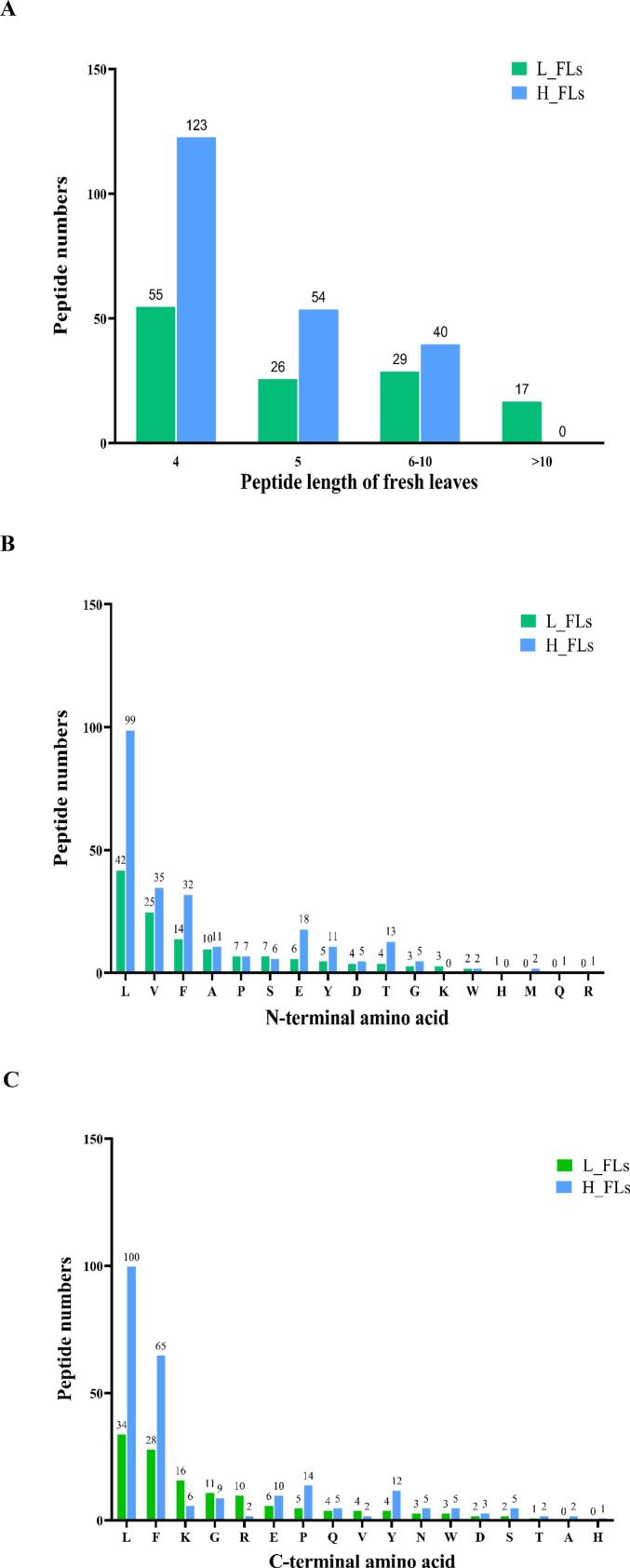
Identification of water-soluble peptides in fresh leaves of ‘Baiye 1’ from low altitude (L_FLs) and high altitude (H_FLs). (A) Total number of peptides identified; (B) Distribution of N-terminal amino acids; (C) Distribution of C-terminal amino acids. Note: L_FLs, low altitude fresh leaves; H_FLs, high altitude fresh leaves.

Water-soluble peptides were identified based on both their accurate molecular weight (MW) and ion fragmentations using a data-dependent-acquisition (DDA) strategy in combination with de novo sequencing. A total of 326 peptides with ≥99 % average local confidence (ALC) scores were identified from FLs from low and high altitudes (Table S2). Among these, 78 peptides were unique to L_FLs, 193 peptides were unique to H_FLs, and 55 peptides were common to both. Their MWs arranged from 392.21 to 2210.10 Da. Peptides were categorised by length into four groups: 4, 5, 6–10, and > 10 amino acids (AAs) (Fig. 1A). Tetrapeptides were the shortest peptides identified in both L_FLs and H_FLs, while the longest peptide in L_FLs contained 20 AAs, compared to a decapeptide as the longest in H_FLs. This finding represents a novel insight, indicating that tetrapeptides and pentapeptides are the dominant water-soluble peptides in fresh albino tea leaves from both altitudes. Furthermore, analysis of AA composition of peptides revealed distinct N-terminal and C-terminal preferences. Leucine (L), valine (V) and phenylalanine (F) were the most common N-terminal residues, while the C-terminal residues were predominated by L, F and lysine (K), accounting for 59 % of all peptides. In contrast, H_FLs exhibited a dominance of L and F residues at the C-terminus, comprising 66 % (Fig. 1B & C).

Significant differences in peptides profiles between L_FLs and H_FLs were presented in Table 1. Among the 55 peptides common to both groups, seven (e.g. FEFL, LALF, EAPKPLV, LPVVG, LTFL, LPLVG, and DWEP) showed higher peak intensities in L_FLs. Conversely, most of these peptides showed higher intensity in H_FLs. For instance, peptides such as FEFL, ELVF, SLFD, TGWFG, AYLL possessed >10^7^ peak areas in H_FLs. Furthermore, the top 10 specific peptides in L_FLs and H_FLs were also illustrated in Table 1. LQTGTYESAVGNAESR (50.27*10^6^), VATVSLPR (17.18*10^6^), LEGHNLDVLEGNEQFLNAAK (16.94*10^6^) were the top three peptides in the low-altitude samples, while LLEPSL (17.56*10^6^), ALVF (7.66*10^6^), and MQLF (6.06*10^6^) were the top three peptides in the high-altitude samples. It was reasonable to speculate that different cultivation environments significantly affected the content and composition of water-soluble peptides, which aligned with the differential accumulated patterns of secondary metabolites reported in others reports (Ahmed et al., 2019; Kfoury et al., 2018; Sun et al., 2024). The water-soluble peptides in fresh leaves at high altitude are more abundant than those in fresh leaves at low altitude, because high altitude areas are usually characterized by more rainfall, continuous cloud cover and increased diffuse radiation (especially in the blue-purple wavelength range), which may inhibit protein synthesis and promote proteolytic enzyme activity, leading to the decomposition of protein into small molecular peptides, thus enhancing the stability and accumulation of peptides (Wang et al., 2022). Nevertheless, the exact regulatory mechanisms behind altitude-induced peptide variation remain complex and merit further investigation. Previous studies have demonstrated that oligopeptides contribute substantially to taste perception, particularly umami, bitterness, and sweetness (Kumar et al., 2023). Thus, the differential accumulation of water-soluble peptides in FLs at different altitudes may directly contribute to the perceived richness and complexity of tea infusion taste. And it was worth carrying out sensory evaluation of single peptides according to previous study (Pu et al., 2023; Shan et al., 2022).

**Table 1:** Water-soluble peptide sequences in ‘Baiye 1’ fresh leaves collected from low and high altitudes.

Peptide sequence^a^	Peak area (10^6^) ^b^		Length	Mass	Flavor prediction ^c^
	Low	High			
**Common peptides identified in both altitudes**					
FEFL	55.09	52.63	4	554.274	/
LALF	5.52	1.43	4	462.2842	Bitter (0.56)
EAPKPLV	3.26	1.48	7	752.4432	Umami (0.54)
LPVVG	1.52	0.31	5	483.3057	Bitter (0.61)
LTFL	1.17	1.13	4	492.2948	/
LPLVG	1.04	0.43	5	497.3213	/
DPWEP	0.32	0.19	5	642.2649	Bitter (0.72)
ELVF	5.85	20.29	4	506.274	Bitter (0.84)
VGFTF	5.43	7.26	5	569.2849	/
SLFD	5.41	18.51	4	480.222	Bitter (0.57)
PLNRLAVG	5.01	9.11	8	838.5024	Bitter (0.98)
TGWFG	4.45	11.11	5	566.2489	/
AYLL	2.70	10.21	4	478.2791	Bitter (0.54)
FLAL	2.15	9.84	4	462.2842	Bitter (0.57)
GPLW	1.96	2.95	4	471.2481	Bitter (0.69)
APLPLG	1.71	3.24	6	566.3428	/
LSLF	1.68	3.71	4	478.2791	/
LEPGPLS	1.59	2.39	7	711.3802	/
LLSL	1.52	5.12	4	444.2948	/
LVGANLL	1.35	2.07	7	698.4327	Bitter (0.99)
LLNPLS	1.34	2.30	6	655.3904	Bitter (0.73)
FTTF	1.30	4.85	4	514.2427	/
VVLF	1.27	3.81	4	476.2998	/
TLLF	1.27	2.21	4	492.2948	/
LPVGVY	1.21	2.69	6	646.369	/
LALL	1.11	3.74	4	428.2998	Bitter (0.97)
VVLL	1.03	3.41	4	442.3155	/
AAYLL	0.98	2.00	5	549.3162	Bitter (0.95)
FLDNF	0.97	3.98	5	654.3013	/
VLEL	0.86	3.71	4	472.2897	Umami (0.66)
LLTF	0.79	2.33	4	492.2948	/
SVGPF	0.74	2.04	5	505.2536	Bitter (0.55)
SPFL	0.72	2.03	4	462.2478	/
FGAF	0.70	1.99	4	440.206	Bitter (1)
FTLE	0.68	1.99	4	508.2533	Bitter (0.58)
VLLDL	0.67	1.08	5	571.3581	Bitter (0.7)
VVLTTL	0.66	1.91	6	644.4109	/
LPLE	0.61	1.25	4	470.274	Bitter (0.5)
VVVL	0.57	1.30	4	428.2998	Bitter (0.87)
LSLL	0.56	1.36	4	444.2948	Bitter (0.54)
YFSAL	0.53	4.01	5	599.2955	/
VVMGF	0.53	2.90	5	551.2777	/
WAFE	0.52	2.49	4	551.238	/
YEVW	0.50	1.20	4	595.2642	/
LLDF	0.47	2.21	4	506.274	/
PDWP	0.47	0.49	4	513.2224	/
LTVL	0.45	2.16	4	444.2948	Umami (0.53)
LLAL	0.43	1.64	4	428.2998	Bitter (0.8)
LTLL	0.40	1.34	4	458.3104	/
LVSL	0.38	2.88	4	430.2791	/
LSVF	0.29	1.53	4	464.2635	/
VLLP	0.29	2.20	4	440.2998	/
LSVL	0.15	0.56	4	430.2791	/
LPLQ	0.13	0.42	4	469.29	/
VVVF	0.10	0.36	4	462.2842	Bitter (0.85)

**Specific peptide in low altitude (Top 10)**^**d**^					
LQTGTYESAVGNAESR	50.27	0	16	1681.79	Bitter (0.82) Umami (0.68)
VATVSLPR	17.18	0	8	841.50	Umami (0.73)
LEGHNLDVLEGNEQFLNAAK	16.94	0	20	2210.10	Bitter (0.93) Umami (0.56)
EESSSKSSQPLASKQEK	16.32	0	17	1848.91	/
APPTP	14.90	0	5	481.25	/
LSAKPAPPKPEKPPK	14.58	0	15	1583.94	Bitter (1)
LSAKPAPPKPEPKPK	14.58	0	15	1583.94	Bitter (1)
SSFDLKPVN	13.45	0	9	1005.51	/
LSAKPAPPKPEPKPR	9.44	0	15	1611.95	Bitter (1)
KGSKAPPPKPEPKPK	8.74	0	15	1584.94	Bitter (1)

**Specific peptide in high altitude (Top 10)**^**e**^					
LLEPSL	0	17.56	6	670.39	/
ALVF	0	7.66	4	448.27	Bitter (0.85)
MQLF	0	6.06	4	537.26	/
GVFTF	0	5.43	5	569.28	/
TFTF	0	4.85	4	514.24	/
EQTVVLK	0	4.57	7	815.48	Umami (0.6)
FSLL	0	3.78	4	478.28	/
FEGGF	0	3.70	5	555.23	Bitter (0.83)
FENF	0	3.68	4	555.23	Bitter (0.58)
LVLP	0	3.66	4	440.30	/

Note:a, peptides detailed information were listed in Table S1.b，peak areas presented as the results of mean value of three replicates and SD was calculated within 0.00–0.52.c, umami and bitter taste flavor of the selected peptides were predicted according to online database (https://nepc2pvmzy.us-east-1.awsapprunner.com/), and these active ones were proceeded Alphafold 3 modeling with T1R1/T1R3; d. ten peptides with the highest peak-area abundance in fresh leaves at low altitude. e. ten peptides with the highest peak-area abundance in fresh leaves at high altitude.

### 3.2 Differential accumulation of predicted umami and bitter taste peptides during green tea processing from low and high altitudes ‘Baiye 1’

It is well acknowledged that green tea products prepared from ‘Baiye 1’ possess a charming umami and mellow taste compared to other green teas. According to previous studies, the higher AAs, especially theanine, could be the major reason (Chen et al., 2024; Cheng et al., 2019; Yu et al., 2023; Zeng et al., 2024). A novel aspect of our study was that flavor peptides were effectively predicted, and their differential accumulation and change patterns during GT processing were further unraveled.

**Fig. 2:**
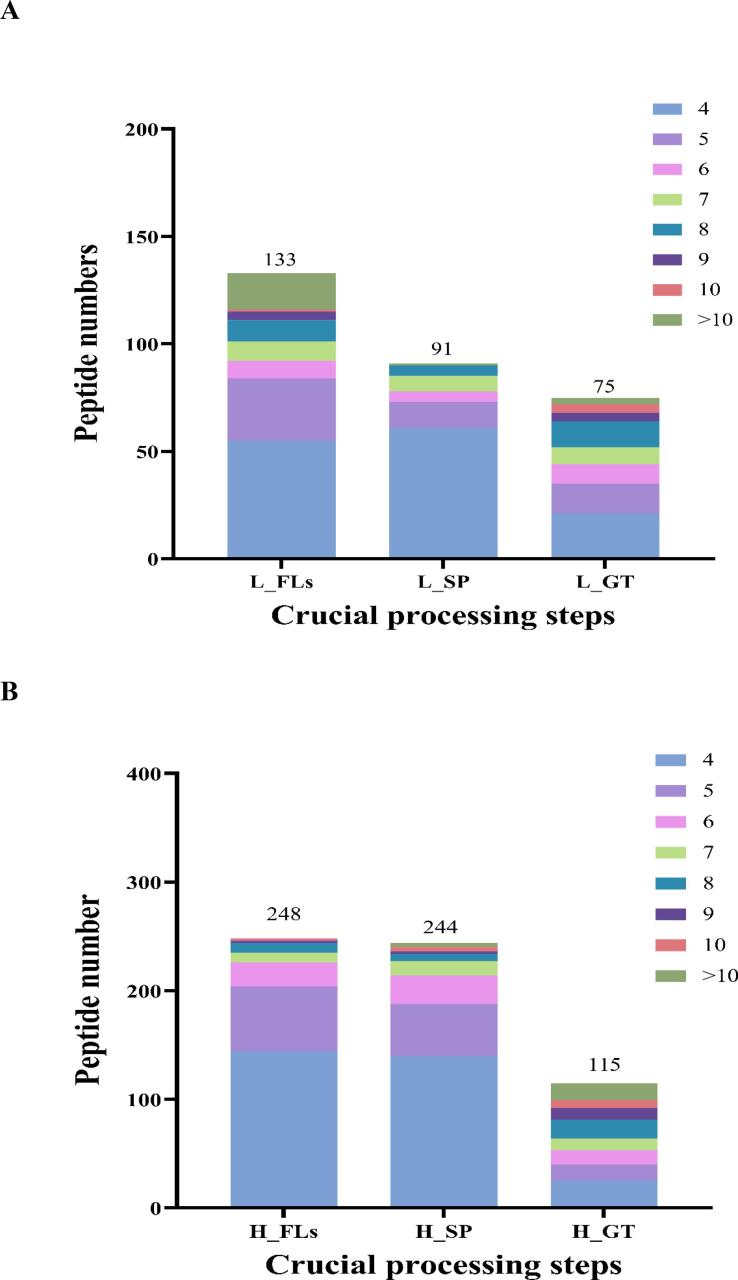

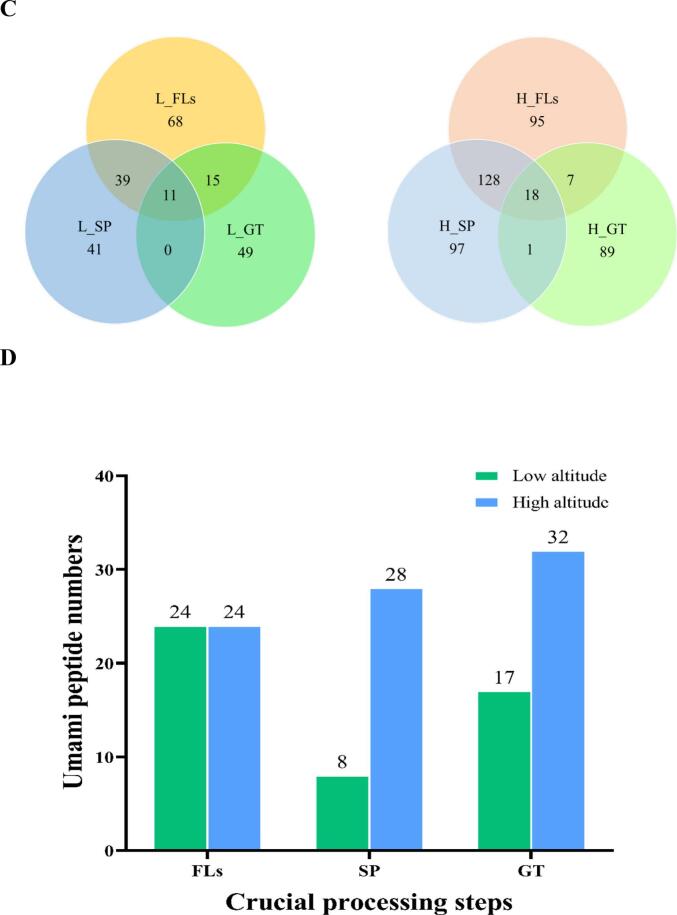

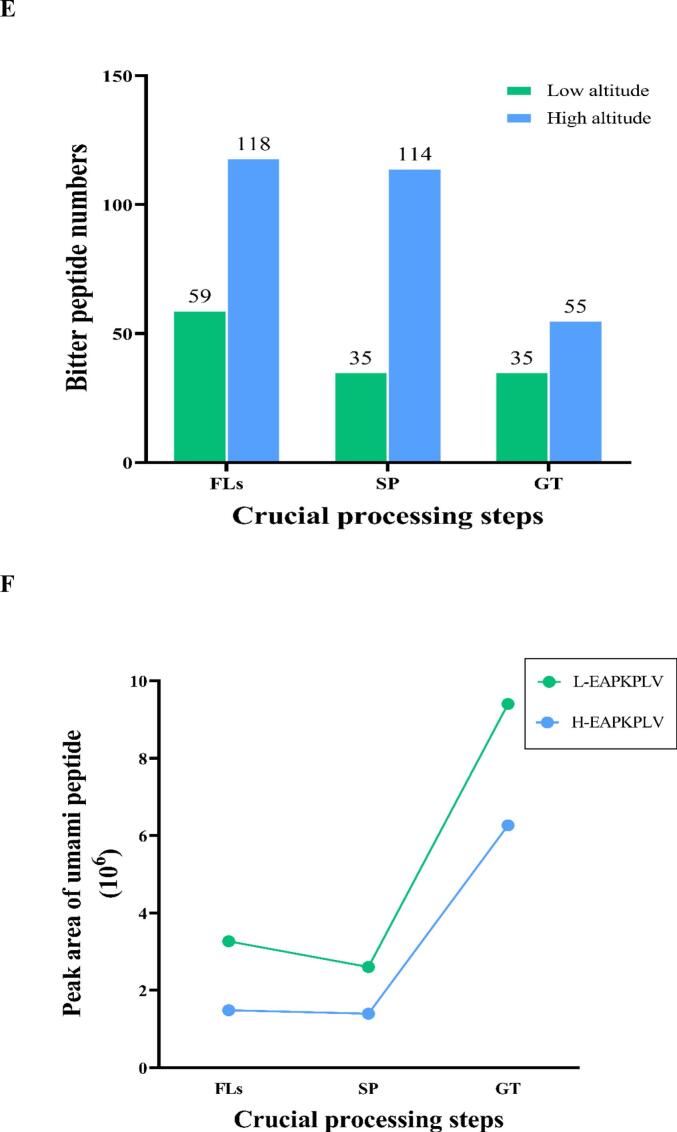

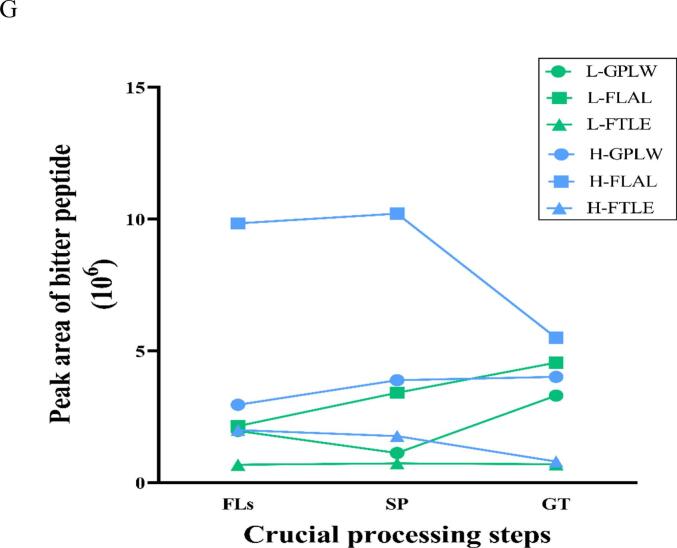
Differential accumulation of peptides during green tea processing of ‘Baiye 1’ from low and high altitudes. (A) Peptide number and length distribution during processing (low altitude); (B) Peptide number and length distribution during processing (high altitude); (C) Venn diagram showing overlap of peptide identifications; (D) Number of flavor peptides during processing (low altitude); (E) Number of flavor peptides during processing (high altitude); (F) Changes in peak areas of common umami peptides; (G) Changes in peak areas of common bitter peptides. Note: L_FLs, low altitude fresh leaves; L_SP, low altitude spreading step; L_GT, low altitude green tea; H_FLs, high altitude fresh leaves. H_SP, high altitude spreading step; H_GT, high altitude green tea. L-EAPKPLV, low altitude-EAPKPLV; H-EAPKPLV, high altitude-EAPKPLLV; L-FLAL, low altitude- FLAL; L-FTLE, low altitude- FTLE; L-GPLW: low altitude- GPLW; H-FLAL, high altitude-FLAL; H-FTLE; high altitude-FTLE; H-GPLW, high altitude-GPLW. (For interpretation of the references to colour in this figure legend, the reader is referred to the web version of this article.)

A total of 299 peptides, ranging in length from 4 to 20 AAs, were identified from low altitude, and 11 peptides were detected in L_FLs, L_SP and L_GT (Fig. 2 A). A decreasing tendency in peptides was observed from 133 in L_FLs, to 91 in L_SP and 75 in L_GT during continuous processing. As shown in Fig. 2B, there was a different behavior of peptide accumulation in high-altitude samples compared to low-altitude samples as aforementioned that short wave light (mainly blue purple light) of high-altitudes could be a primer to activate more peptides for resistance. However, it needed further investigation and verifying. A total of 607 peptides were identified, and 18 peptides were detected in H_FLs, H_SP and H_GT. The results showed that the H_SP samples from high altitude retained an almost similar number of peptides as H_ FL, while the number was significantly reduced to 115 in the manufactured green tea product (H_GT). Similar to the low-altitude samples, tetrapeptides and pentapeptides were the majority in both GT products. GT processing resulted in significant differences when comparing specific peptides detected in each step, as shown in Fig. 2C. 41 and 97 specific peptides were screened in L_ SP and H_ SP, respectively. Similarly, 49 and 89 specific peptides were identified in L_ GT and H_ GT.

To achieve a further understanding of peptide potential flavor contributions, mainly umami and bitter taste, and their bioactivities (antioxidant, antihypertensive, DPP-IV, and neurotransmitters activity), were predicted using UniDL4BioPep (https://nepc2pvmzy.us-east-1.awsapprunner.com/) (Table S3) (Du et al., 2023). In low-altitude samples, 24 potential umami peptides were predicted in L_FLs, but only 8 were predicted in L_SP and 17 in L_GT samples. In contrast, high-altitude samples had 24 predicted umami peptides in H_FLs, with the numbers increasing to 28 and 32 in H_SP and H_GT, respectively (Fig. 2D). Although more bitter peptides were predicted, similar results were observed, showing a decrease in peptide numbers during continuous processing, as illustrated in Fig. 2E. Although the types of peptides may have changed due to potential degradation by proteases or peptidases, some peptides remain present throughout the entire processing of green tea and are relatively stable compared to most peptides, able to withstand digestion by proteases or peptidases. For example, four flavor peptides, EAPKPLV (umami peptide) and three bitter peptides (FLAL, FTLE, and GPLW), are always present throughout the entire processing process at low and high altitudes. In addition, five bitter peptides, ELFF, FEEF, FLAL, FTLE, and LPLE, were consistently detected throughout the entire processing process in high-altitude areas.

Relative quantification analysis showed that all four peptides, EAPKPLV, FLAL, FTLE, and GPLW, changed significantly during processing at both altitude samples. The peak area of the predicted umami peptide EAPKPLV decreased during spreading (SP) but exhibited a significant increase in the later stages of processing (Fig. 2F). Previous studies have indicated that peptidase activity rises under dehydration stress during spreading, leading to extensive enzymatic degradation of endogenous peptides. Specifically, sites containing Pro and Ser are more susceptible to cleavage, consequently resulting in a reduction of EAPKPLV during spreading (McGrath et al., 2016). In the thermal processing process (fixation and drying), high temperature heating induces thermal denaturation of proteins in tea leaves, exposing more enzyme cleavage sites, and directly breaking peptide bonds without complete inactivation or thermal action to regenerate EAPKPLV. (Davies, 2005). The predicted bitter peptide trends are more complex, as shown in Fig. 2G. At low altitude, L-FLAL accumulated continuously with processing, while L-FTLE increased during SP and decreased significantly at late processing. L-GPLW showed the opposite trend. At high altitude, the peak area of H-FLAL increased during spreading and decreased significantly at the later stage of processing; the peak area of H-FTLE decreased gradually with processing, while the peak area of H-GPLW accumulated continuously with processing. This complex trend is due to the balance between enzymatic degradation and synthesis during processing: peptidase activity increases during spreading due to dehydration stress, resulting in a decrease in some water-soluble peptides (e.g. L-GPLW, H-FTLE) during withering (Kottur et al., 2011); however, some enzymes involved in protein degradation (ATP-dependent Clp protease proteolysis subunit 4) are highly expressed, resulting in an increase in some short peptides (e.g. L-FLAL, L-FTLE) during spreading (Ni et al., 2021; Wu et al., 2018)). In the thermal processing stage (de-enzyming and drying), heat effect causes protein denaturation to expose enzyme cleavage sites, thermostable protease action and direct thermal decomposition promote peptide production (e.g. L-FLAL, L-GPLW) (Ebner et al., 2016); but at the same time, Maillard reaction between peptide and sugar occurs under high temperature conditions, thus consuming part of peptide (e.g. H-FLAL, H-FTLE) (Fu et al., 2020). These results further verified that processing promoted the production of umami peptides, providing new insights into umami and mellow flavor characteristic of GT.

### 3.3 Differences of amino acids in ‘Baiye 1’ harvested from low and high altitudes and their alteration during GT processing

**Fig. 3:**
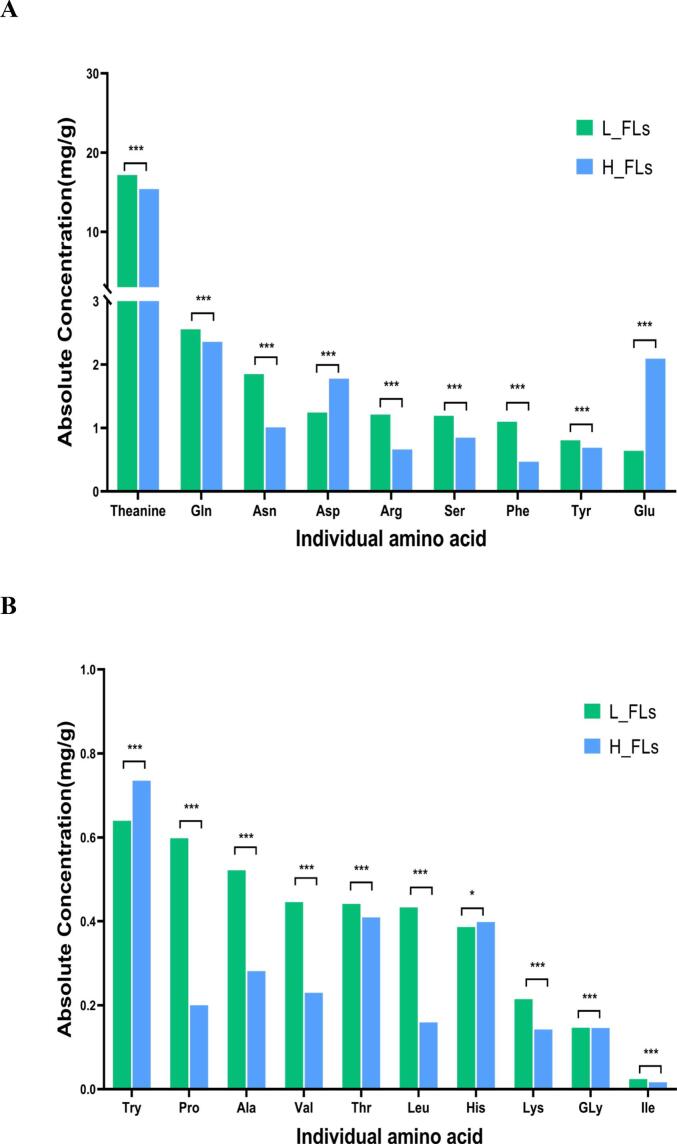

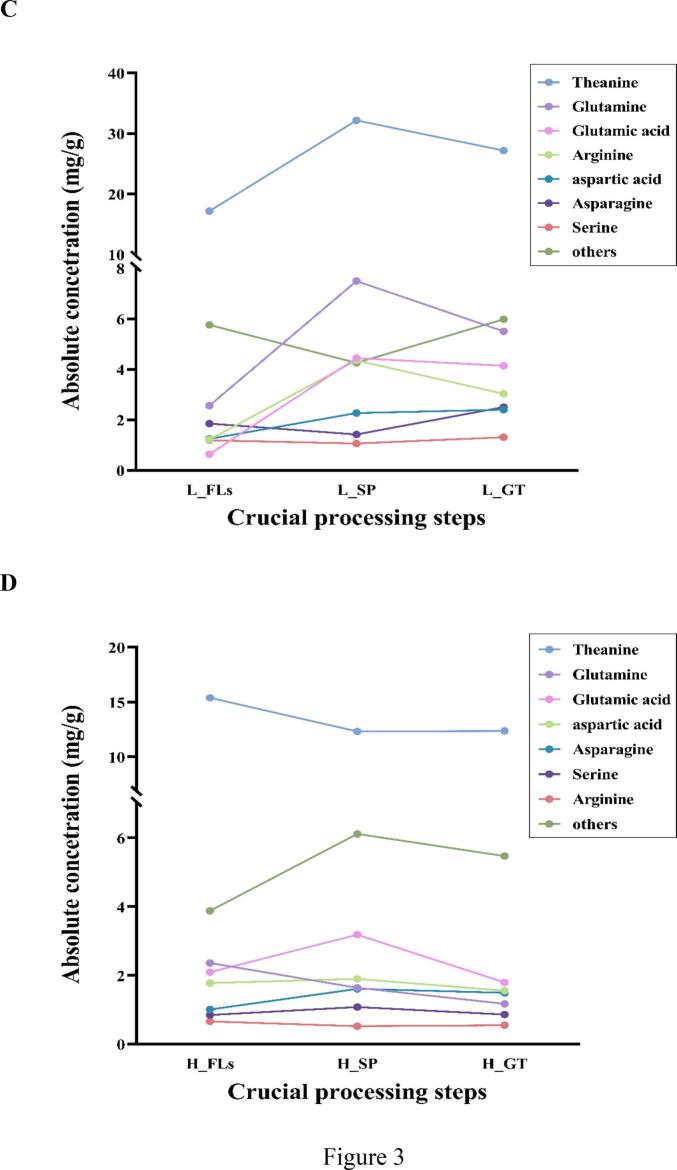
Concentrations and dynamic changes of major amino acids during green tea processing. (A) and (B) Amino acid concentrations in fresh leaves from low and high altitudes; (C) Changes in amino acids during processing (low altitude); (D) Changes in amino acids during processing (high altitude). Note: L_FLs, low altitude fresh leaves; L_SP, low altitude spreading step; L_GT, low altitude green tea; H_FLs, high altitude fresh leaves. H_SP, high altitude spreading step; H_GT, high altitude green tea. Statistical comparisons were based on differences between low and high altitude (ns = not significant, * = Significant at *P* < 0.05 level, *** = Significant at 0.01 < *P* < 0.001 level); (For interpretation of the references to colour in this figure legend, the reader is referred to the web version of this article.)

Targeted metabolomics profiling of AAs provides important insights into the flavor-active compounds present in ‘Baiye 1’ and their variation during green tea processing (Table S4). A total of 18 essential AAs and theanine were quantitatively analyzed in ‘Baiye 1’ FLs collected from different altitudes (Fig. 3A and B). In low-attitude FLs, the total AAs content was 31.62 ± 0.30 mg/g, with theanine being the most abundant, accounting for 54 % (17.17 ± 0.16 mg/g). Glutamine, asparagine, and aspartic acid- three key contributors to umami taste-followed, with respective concentrations of 2.56 ± 0.02, 1.85 ± 0.02, 1.25 ± 0.01 mg/g. In contrast, in high-altitude FLs exhibited a total AAs content was 28.01 ± 0.27 mg/g, with theanine at 15.39 ± 0.15 mg/g. Glutamine, glutamic acid, and asparagine were the next abundant, at 2.36 ± 0.02, 2.09 ± 0.02, and 2.09 ± 0.02 mg/g, respectively. Notably, isoleucine was the least abundant AA in both groups, with 0.024 ± 0.00 mg/g in L_FLs and 0.016 ± 0.00 mg/g in H_FLs. In total, H_FLs showed an 11 % reduction in AA content compared to L_FLs. Thirteen Aas-including theanine, glutamine, asparagine, arginine and serine- were significantly reduced in H_FLs by 10.36 %, 7.72 %, 45.51 %, 45.62 % and 28.87 %, respectively. Conversely, aspartic acid, glutamic acid, histidine and tryptophan increased by 14.98 %, 226.89 %, 42.59 %, and 3.11 %, respectively. Previous studies have indicated that altitude significantly impacts the biosynthesis of nitrogenous compounds, including AA (Han et al., 2017). High-altitude environments, characterized by lower temperatures, higher humidity, and frequent fog, favour the accumulation of glutamate and related nitrogenous metabolites (Tian et al., 2024). However, the abundant nitrogen availability in low-altitude tea gardens may contribute more effectively to the biosynthesis of theanine and glutamine (Dong et al., 2019). Indeed, L-theanine synthetase (CsTSI), which is highly homologous to glutamine synthetase (CsGS), catalyses the formation of both l-glutamine and L-theanine from glutamate. This enzymatic activity is significantly promoted by nitrogen fertilization (Li et al., 2024). The reduced levels of theanine and glutamine, alongside the elevated glutamate concentration observed in H_FLs, are consistent with the downregulation of the CsTS and CsGS genes and the upregulation of the L-theanine degradation gene (CsPDX2.1) under high-altitude conditions (Wang et al., 2022).

Alterations in AAs during GT processing at low altitude are shown in Fig. 3C. Total AAs increased from 31.62 mg/g in L_FLs to 52.05 mg/g in L_GT, primarily due to theanine, glutamic acid, glutamine, and arginine, which significantly increased in L_SP and maintained at higher levels in L_GT. Besides, aspartic acid, serine, and leucine also increased in L_GT. In contrast, AAs in high-altitude samples slightly declined during GT processing, from 28.01 mg/g in H_FLs to 25.25 mg/g in H_GT. Most umami-related AAs—including theanine, glutamic acid, and arginine—decreased gradually, whereas aspartic acid slightly increased (Fig. 3D). Theanine, glutamic acid, aspartic acid, and glutamine are all well recognized for contributing to umami flavor; glycine, alanine, glutamine, threonine, and serine contribute sweetness; and histidine, valine, and tryptophan are linked to bitterness (Yu & Yang,2020). It is worth noting that most AAs increased during the spreading process, where protease and peptidase activities were triggered by water-loss (Qiao et al., 2023). However, the differential accumulation patterns observed during processing could be complex, influenced by cultivation conditions, including heavy fertilization, as discussed in our previous study. The higher levels of umami AAs (theanine, glutamic acid, and glutamine) and sweetness AA (serine and threonine) in L_GT compared to H_GT could explain the umami and mellow flavor in low altitude cultivated teas.

### 3.4 Differential accumulation of major pigments (carotenoids and flavonoids) in FLs harvested from low and high altitudes

A total of 22 carotenoids were quantified, including 3 carotenes and 19 xanthophylls (Table 2; Table S5). In L_FLs, the total carotene content reached 39.07 ± 0.37 μg/g, with β-carotene being the most abundant at 30.98 ± 0.29 μg/g. The total xanthophylls content was 377.29 ± 3.59 μg/g, with lutein having the highest concentration at 350.80 ± 3.34 μg/g, followed by zeaxanthin at 15.77 ± 0.15 μg/g, neoxanthin at 6.05 ± 0.06 μg/g, β-cryptoxanthin at 2.21 ± 0.02 μg/g, and violaxanthin at 1.28 ± 0.01 μg/g. Other xanthophylls, such as α-cryptoxanthin, lutein palmitate, lutein myristate, and lutein dimyristate, were present at very low concentrations in L_FLs (<0.5 μg/g). Environmental factors, particularly temperature and light, play key roles in the accumulation of carotenoids, and altitude is typically negatively correlated with temperature (Ahmed et al., 2019). The results were in accordance with this finding that the total carotene and lutein in H_FLs (14.83 ± 0.14 μg/g and 178.24 ± 1.70 μg/g, respectively) were significantly lower than in L_FLs. β-Carotene remained the most abundant carotene at 12.62 ± 0.12 μg/g, while (E/Z)-phytoene was the least abundant at 0.88 ± 0.01 μg/g. Similarly, lutein was the most abundant in H_FLs at 164.01 ± 1.56 μg/g, followed by zeaxanthin at 9.14 ± 0.09 μg/g and neoxanthin and β-cryptoxanthin at 2.20 ± 0.02 and 1.48 ± 0.01 μg/g, respectively. Other xanthophylls such as α-cryptoxanthin, lutein palmitate, lutein myristate, and lutein dimyristate, were also present at very low concentrations in H_FLs (<0.5 μg/g). Carotenoids, which are yellow to orange-red fat-soluble pigments, serve as important colorants and health-related nutrients, including carotenes and lutein (Shi, Yang, et al., 2023). Previous studies have demonstrated that the expression of carotenoid cleavage dioxygenase 4 increased under low-temperature conditions, contributing to the degradation of β-carotene to β-violet ketone, which reduces β-carotene content (Wang et al., 2020). Yang et al. (2021) also reported that the expression of the VDE genes involved in the biosynthesis of xanthophyll cycle pigments is suppressed in albino tea cultivar at low temperatures, leading to a decrease in xanthophyll content.

**Table 2:** Quantitative analysis of major pigments (carotenoids and flavonoids) in ‘Baiye 1’ fresh leaves at two altitudes.

Compound	Contend	
	Low altitude	High altitude
**Carotenoid(ug·g**^**−1**^**)**		
β-Carotene(C1)	30.98 ± 0.29**	12.62 ± 0.12
α-Carotene(C2)	5.70 ± 0.05**	1.34 ± 0.01
(E/Z)-Phytoene(C3)	2.38 ± 0.02**	0.88 ± 0.01
Lutein(X1)	350.80 ± 3.34**	164.01 ± 1.56
Zeaxanthin(X2)	15.77 ± 0.15**	9.14 ± 0.09
Neoxanthin(X3)	6.05 ± 0.06**	2.20 ± 0.02
β-Cryptoxanthin(X4)	2.21 ± 0.02*	1.48 ± 0.01
Violaxanthin(X5)	1.28 ± 0.01*	0.67 ± 0.01
α-Cryptoxanthin(X6)	0.45 ± 0.00**	0.15 ± 0.00
Lutein palmitate(X7)	0.22 ± 0.00	0.12 ± 0.00
Lutein myristate(X8)	0.18 ± 0.00	0.17 ± 0.00
Lutein dimyristate(X9)	0.07 ± 0.00	0.05 ± 0.00
Lutein dilaurate(X10)	0.07 ± 0.00	0.04 ± 0.00
8′-Apo-beta-carotenal(X11)	0.04 ± 0.00	0.02 ± 0.00
Zeaxanthin dimyristate(X12)	0.03 ± 0.00	0.02 ± 0.00
Lutein oleate(X13)	0.03 ± 0.00	0.01 ± 0.00
Echinenone(X14)	0.03 ± 0.00	0.01 ± 0.00
Zeaxanthin dipalmitate(X15)	0.02 ± 0.00	0.03 ± 0.00
Lutein caprate(X16)	0.02 ± 0.00	0.02 ± 0.00
Violaxanthin dibutyrate(X17)	0.01 ± 0.00	0.03 ± 0.00
β-Citraurin(X18)	0.01 ± 0.00	0.00 ± 0.00
Violaxanthin palmitate(X19)	0.00 ± 0.00	0.07 ± 0.00

**Flavonoids(mg·g**^**−1**^**)**		
EGCG	65.18 ± 0.62**	58.66 ± 0.56
EGC	41.10 ± 0.39	44.12 ± 0.42
ECG	32.83 ± 0.31**	25.40 ± 0.24
EC	16.30 ± 0.16**	12.76 ± 0.12
GC	5.09 ± 0.05*	3.35 ± 0.03
C	2.94 ± 0.03*	2.15 ± 0.02
GCG	0.35 ± 0.00	0.46 ± 0.00**
CG	0.03 ± 0.00	0.04 ± 0.00
M-3-gal	2.98 ± 0.03	2.59 ± 0.02
Q-3-gal	0.83 ± 0.01*	0.64 ± 0.01
M-3′-glu	0.39 ± 0.00	0.52 ± 0.00**
K-3-rut	0.35 ± 0.00	0.47 ± 0.00*
Q-3-glu	0.20 ± 0.00	0.31 ± 0.00**
Myricetin	0.05 ± 0.00	0.07 ± 0.00

Note: Mean values listed in the table were all in three replicates, (x ± SD, *n* = 3). Statistical comparisons were based on differences between low and high altitude (ns = not significant, ** = Significant at *P* < 0.01 level); Low altitude = ∼80 m, high altitude = ∼ 500 m.

In addition to carotenoids, major water-soluble pigments were also assessed, including eight flavan-3-ols (catechins) and six flavonol glycosides (Table 2; Table S6). In L_FLs, the total flavonoid content was 168.61 ± 1.60 mg/g, with catechins accounting for 163.82 ± 1.56 mg/g. Epigallocatechin gallate (EGCG) was the most abundant individual compound at 65.18 ± 0.62 mg/g, comprising 39.79 % of total catechins, followed by EGC (41.10 ± 0.39 mg/g), ECG (32.83 ± 0.31 mg/g), and EC (16.30 ± 0.16 mg/g). Gallocatechin (GC) and catechin (C) were present at 5.09 ± 0.05 and 2.15 ± 0.02 mg/g, respectively, while GCG and CG were detected in lower amounts (0.35 ± 0.00 and 0.03 ± 0.00 mg/g, respectively). Among flavonol glycosides, M-3-gal was the most abundant (2.98 ± 0.03 mg/g), followed by Q-3-gal (0.83 ± 0.01 mg/g), with others such as M-3′-glu, K-3-rut, Q-3-glu, and myricetin present at <0.5 mg/g.

The flavonoid content was lower in H_FLs, with total flavonoids measured at 151.56 ± 1.44 mg/g and catechin concentrations at 146.95 ± 1.40 mg/g. Major catechins like EGCG (58.66 ± 0.56 mg/g) accounted for 39.92 % of total catechins, with EGC, ECG, and EC all exceeding 10 mg/g. GCG and CG were present in low concentrations, with values of 0.46 ± 0.00 mg/g and 0.04 ± 0.00 mg/g, respectively. The total flavonol and glycoside content was 4.61 ± 0.04 mg/g, with M-3-gal at 2.59 ± 0.02 mg/g and M-3′-glu at 0.64 ± 0.01 mg/g. Other flavonols like K-3-rut, Q-3-glu, and myricetin were present at very low concentrations (<1 mg/g).

Catechins are the primary flavonoids in tea and are closely associated with bitterness and astringency (Ye et al., 2022). Prior studies have demonstrated that EGCG and ECG levels tend to decrease with increasing elevation (Ye et al., 2023; Han et al., 2017), which aligns with the present findings. Moreover, the total catechin and flavonoid glycoside contents in H_FLs were significantly lower by 10.3 % and 3.89 %, respectively, particularly for compounds like EGCG, ECG, EC, GC, C, M-3-gal, and Q-3-gal, which demonstrated a significant negative correlation with altitude. Supporting these findings, previous studies have indicated that the expression of catechin biosynthesis-related genes (CsDFR1, CsANS2, and CsANR1) is down-regulated as altitude increases (Wang et al., 2020a). However, due to the complexities of different cultivars and cultivation environmental conditions, the variations in other catechins were not entirely consistent with results from previous studies. Flavonol glycosides in tea not only produce a dry and smooth astringent flavor but also resulted in sweet aftertaste (Shi et al., 2023). The relatively higher flavonol glycoside levels in L_FLs may result from enhanced carbohydrate accumulation and upregulation of flavonol biosynthetic pathways under nitrogen-rich conditions common in low-altitude cultivation (Dong et al., 2019).

### 3.5 Dynamic changes of carotenoids and flavonoids during GT processing

**Fig. 4:**
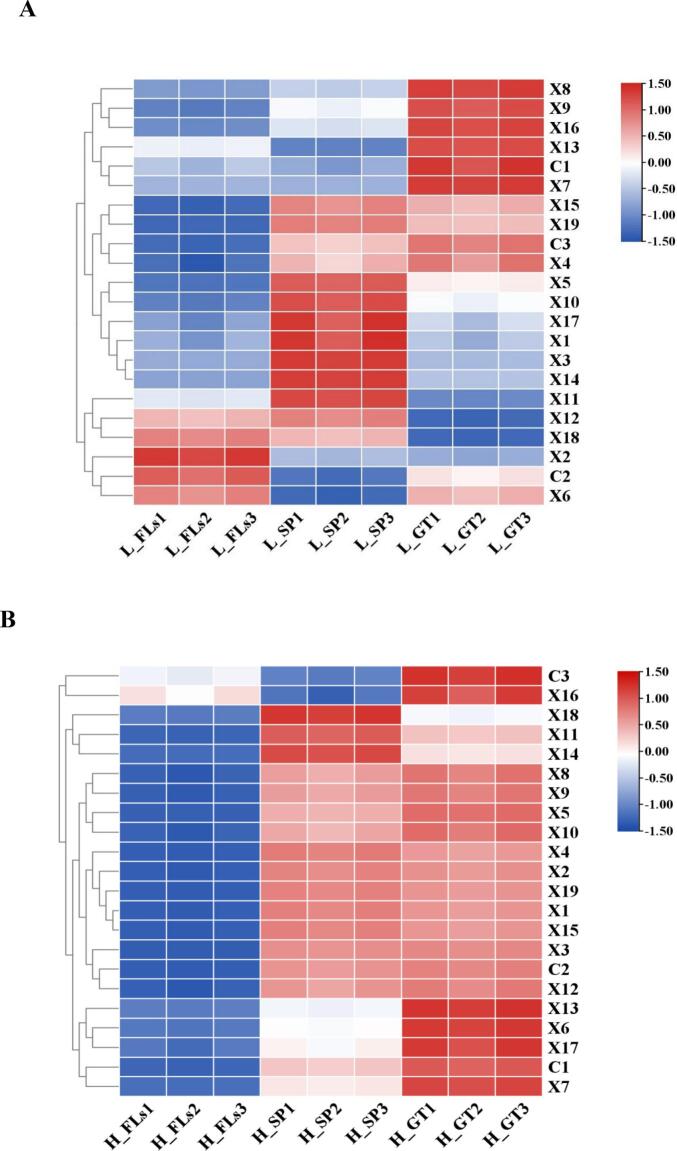

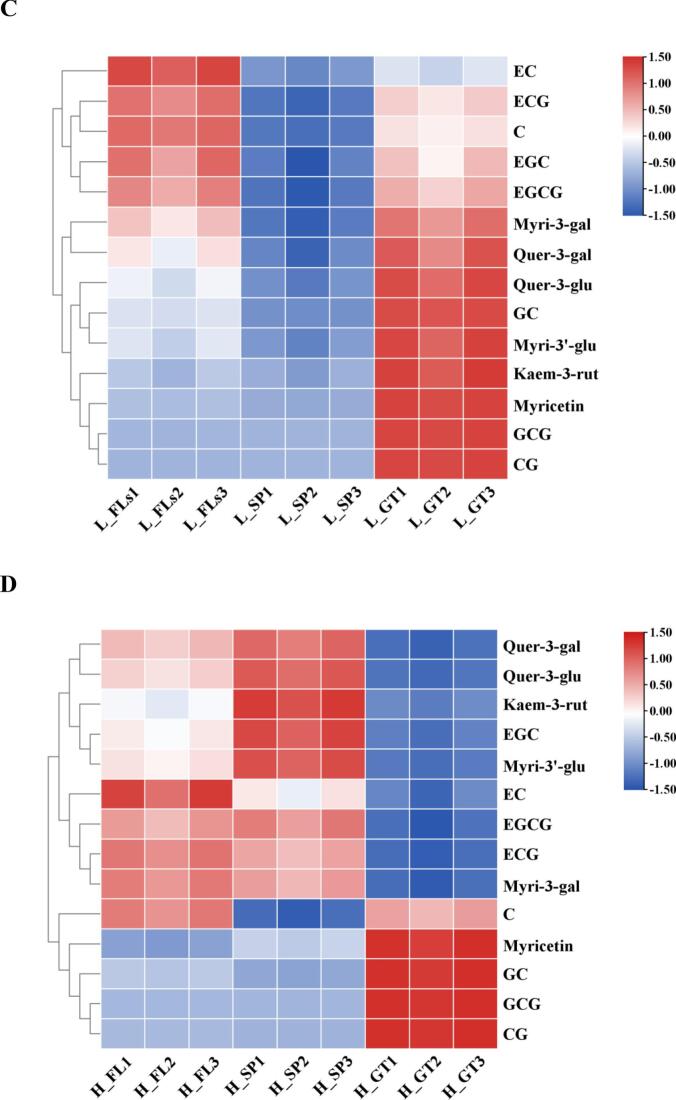
Dynamic changes of carotenoids and flavonoids during GT processing from low altitude and high altitude of ‘Baiye 1’. (A) Dynamic changes of carotenoids during GT processing from low altitude of ‘Baiye 1’. (B) Dynamic changes of carotenoids during GT processing from l high altitude of ‘Baiye 1’. (C) Dynamic changes of flavonoids during GT processing from low altitude of ‘Baiye 1’. (D) Dynamic changes of flavonoids during GT processing from high altitude of ‘Baiye 1’. Note: L_FLs, low altitude fresh leaves; L_SP, low altitude spreading step; L_GT, low altitude green tea; H_FLs, high altitude fresh leaves. H_SP, high altitude spreading step; H_GT, high altitude green tea. (For interpretation of the references to colour in this figure legend, the reader is referred to the web version of this article.)

Green tea (GT) processing led to significant dynamic changes in both carotenoid and flavonoid contents in ‘Baiye 1’ leaves. The alterations observed during processing were found to differ notably between low- and high-altitude samples. As illustrated in Fig. 4A, total carotenoid content increased notably during spreading to 468.43 μg/g but decreased in low-altitude green tea (L_GT) to 424.59 μg/g. The contents of β-carotene (C1), (E/Z)-phytoene (C3), β-cryptoxanthin (X4), lutein palmitate (X7), lutein myristate (X8), lutein dimyristate (X9), lutein oleate (X13), and zeaxanthin dipalmitate (X15) were relatively low in L_FL but significantly accumulated after processing in L_GT. In contrast, the contents ofα-carotene (C2), zeaxanthin (X2), α-cryptoxanthin (X6), and zeaxanthin dimyristate (X12) exhibited the opposite trend, being abundant in L_FL but significantly reduced after processing in L_GT. Additionally, the contents of lutein (X1), neoxanthin (X3), violaxanthin (X5), lutein dilaurate (X10), 8′-apo-beta-carotenal (X11), and echinenone (X14) were significantly increased in L_SP but significantly decreased in L_GT. Notably, the accumulation patterns of individual carotenoids in H_GT differed significantly from those in L_GT (Fig. 4 B). The total carotenoid content in high-altitude samples increased continuously during processing, reaching 428.54 μg/g in H_SP and 422.62 μg/g in H_GT. The content of most carotenoids such as β-carotene (C1), α-carotene (C2), lutein (X1), and zeaxanthin (X2) was low in H_FL, increased significantly in H_SP, and remained high in H_GT.

Carotenoids are not only important pigments but also precursors of key aromatic compounds such as β-ionone and α-ionone. Both enzymatic (e.g., polyphenol oxidases, carotenoid cleavage dioxygenases) and non-enzymatic reactions (e.g., oxidation, isomerisation) occur during GT processing (Li et al., 2011; Wang et al., 2020; Yang et al., 2021; Zhang, Zhang, Pérez-Moreno, Bin, et al., 2024). In the present study, the observed increase in β-carotene during GT processing at both altitudes may be attributed to non-enzymatic condensation of geranylgeranyl pyrophosphate, a carotenoid precursor. Likewise, the rise in lutein levels during the spreading stage could result from enhanced biosynthesis of α-carotene under water loss conditions (Zhang, Zhang, Pérez-Moreno, Bin, et al., 2024).

Systematic exploration of flavonoids dynamics during GT production revealed differences between low altitude and high-altitude samples (Fig. 4C). Total flavonoid content was similar in the L_GT (171.17 mg/g) compared to L_FLs (168.61 mg/g). As the most abundant catechin, EGCG decreased significantly in L_SP (57.67 mg/g) but increased in L_GT (64.17 mg/g), maintaining similar levels to L_FLs (65.18 mg/g). EC, ECG, C and EGC exhibited similar patterns. M-3-gal, Q-3-gal, Q-3-glu, GC, and M-3′-glu increased in L_GT. In contrast, high-altitude green tea processing showed different trends (Fig. 4D) Total flavonoid content remained similar from H_FLs (151.55 mg/g) to H_SP (154.73 mg/g) but decreased in H_GT (138.39 mg/g). EGCG followed a similar trend, remaining steady from FLs (58.66 mg/g) to SP (59.44 mg/g), but decreased significantly in GT (51.34 mg/g). Similar patterns were observed for ECG and M-3-gal. Other flavonoids showed different trends, EGC and four flavonol glycosides increased from H_FLs to H_SP, while EC and C decreased.

Flavonoids are well recognized for their contribution to bitterness (e.g., EGCG, ECG) and astringency (e.g., flavonol glycosides) in tea, and they also contribute to a sweet aftertaste (Zhang, Zhang, Pérez-Moreno, Bin, et al., 2024). In the present study, catechins showed complex trends during staging at both high and low altitudes, which may be attributed to the fact that enzymatic oxidation of catechins and hydrolysis of ester-type catechins occur simultaneously during staging, with catechins slowly oxidized and degraded by polyphenol oxidizing enzymes, and ester-type catechins hydrolyzed to simple catechins (Li et al., 2011). The final flavonoid profiles suggest that L_GT retained more flavonoids overall compared to H_GT. Furthermore, the ratio of non-gallocatechin to gallocatechin was higher in H_GT, which may contribute to a sweeter aftertaste. The observed reduction in flavonol glycosides is likely the result of thermal hydrolysis, which produces saccharides and glycosidic ligands—compounds that may further contribute to the mellow and less bitter character of high-altitude teas (Wang et al., 2021).

### 3.6 Comprehensive analysis of taste-related compounds and their contribution to made GT prepared from ‘Baiye 1′

A comprehensive analysis was undertaken to elucidate the contributions of amino acids, flavonoids, and flavor peptides to the sensory properties of green tea produced from ‘Baiye 1’ harvested at different altitudes. Advanced modeling of umami and bitter taste receptors, facilitated by AlphaFold 3 (https://golgi.sandbox.google.com/fold/268faad69ee34866), enabled in-depth exploration of the binding interactions between these bioactive compounds and taste receptors. The heterodimeric T1R1/T1R3 complex was identified as the principal receptor mediating umami taste, while TAS2R39 was selected as a representative receptor involved in bitterness perception. Due to the relatively simpler structural configuration of T1R1 compared with T1R3, modeling of flavor peptides was primarily conducted with T1R1. In total, seven umami peptide–T1R1 complexes and five bitter peptide–TAS2R39 complexes were constructed under optimised parameters (Fig. S1).

**Fig. 5:**
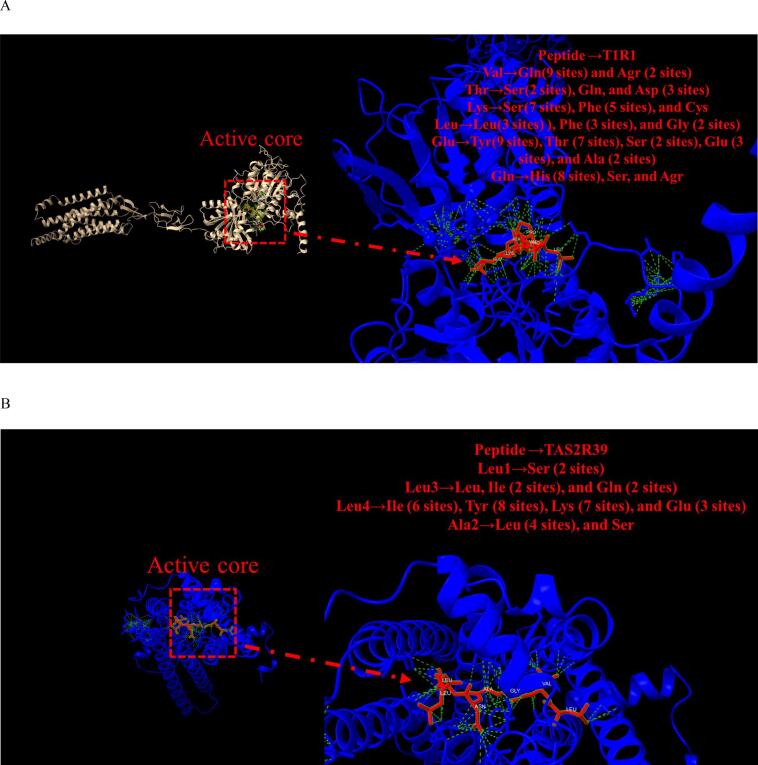

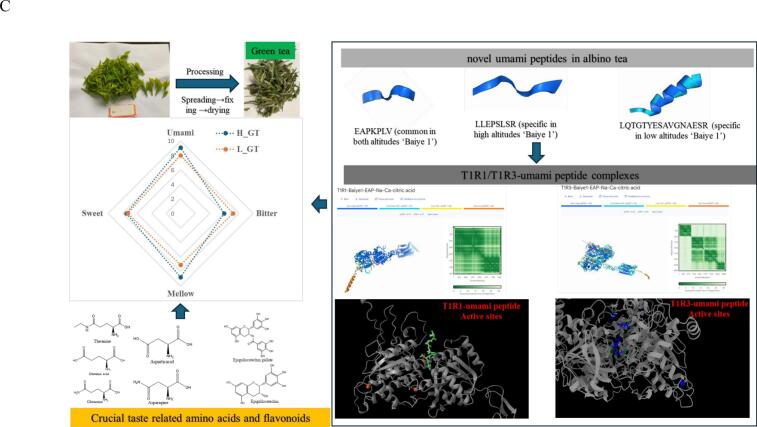
Molecular modeling of novel flavor peptides with taste receptors and proposed mechanism of flavor formation. (A) Predicted complex of EAPKPLV with the T1R1 receptor, bound with citric acid, ATP, Na^+^, and Ca^2+^; (B) Predicted complex of IVGANLL with the TAS2R39 receptor, with the same cofactors; (C) Summary of key insights into the molecular basis of flavor formation in albino tea.

Detailed structural analysis focused on the EAPKPLV–T1R1 umami complex and the LALL–TAS2R39 bitter complex to better understand their respective interaction mechanisms (Fig. 5A & 5B). In the T1R1-EAPKPLV complex, 210 contacts were predicted, including overlapping and H-bond interactions between ligands and the receptor, involving ions (Na^+^ and Ca^2+^), citric acid, and ATP. Specifically, 71 hydrogen bonds were predicted between the umami peptide (valine, threonine, lysine, leucine, glutamine, and glutamic acid) and the T1R1 receptor, with distances ranging from 2 to 4 Å. As illustrated in Fig. 5A, there were multiple binding sites between specific residues: 11 hydrogen bonds between Val and Gln (9) and Arg (2), 6 hydrogen bonds between Thr and Asp (3), Ser (2), and Gln (1), 13 hydrogen bonds between Lys and Ser (7), Phe (5), and Cys (1), 8 hydrogen bonds between Leu and Leu (3), Phe (3), and Gly (2), 10 hydrogen bonds between Gln and His (8), Ser (1), and Arg (1), and 23 hydrogen bonds between Glu and Tyr (9), Thr (7), Glu (3), Ser (2), and Ala (2). For the TAS2R39-LALL bitter complex, a total of 39 binding sites were identified, with distances ranging from 2.1 to 4.1 Å. As shown in Fig. 5B, the binding interactions included 2 hydrogen bonds between Leu1 and Ser (2), 5 hydrogen bonds between Leu3 and Ile (2), Gln (2), and Leu (1), 24 hydrogen bonds between Leu4 and Ile (6), Tyr (8), Lys (7), and Glu (3), and 5 hydrogen bonds between Ala2 and Leu (4) and Ser (1). The precise coordinates and distances for all interactions are detailed in the supplementary material.

By integrating molecular modeling results with compositional analysis (amino acids, flavonoids, and peptides), a more comprehensive understanding of taste development in ‘Baiye 1’ green tea was achieved (Fig. 5C). Studies have shown that umami, mellowness, sweetness, and bitterness are the main taste attributes, and chemical analysis confirmed the pivotal role of specific amino acids—particularly theanine, glutamine, glutamic acid, aspartic acid, and asparagine—in producing the umami and mellow notes. Furthermore, the differential accumulation of flavonoids under varying altitudes and processing conditions, especially EGCG and flavonol glycosides—confirmed their influence on bitterness and astringency. This aligns with existing literature highlighting their critical contributions to tea flavor. (Zhang et al., 2020). The peptide modeling results provided novel insights into the interaction between newly identified peptides and taste receptors. The umami peptide EAPKPLV and bitter peptide LALL exhibited strong, specific binding affinities to T1R1 and TAS2R39, respectively, supporting their potential sensory functions.

These findings offer a multidimensional view of how taste-related compounds—spanning primary metabolites and peptides—work in concert to define the sensory profile of albino green tea. The integration of biochemical, sensory, and receptor interaction data presents a novel contribution to the field of tea flavor chemistry and offers a foundation for future functional characterisation of tea-derived peptides. However, this study only preliminarily explored the distribution characteristics of flavor compounds in tea at different altitudes and during green tea processing. The mechanism of their influence by factors such as altitude and processing technology needs further investigation, and the contribution of water-soluble peptides with potential flavor activity urgently requires further sensory verification.

## 4 Conclusion

Overall, this study provides valuable insights into how altitude and processing influence the flavor profile of albino tea, particularly ‘Baiye 1’. Altitude cultivation differences significantly impacted on the accumulation of umami amino acids (especially theanine, glutamic acid, and asparagine), EGCG and ECG, and the major carotenoids (lutein and β-Carotene). It was newly found >300 flavor related peptides, and that high-altitude environments promoted the accumulation of some umami and bitter peptides. The dynamic changes during processing highlighted the critical role of the spreading and drying steps in modulating and reshaping green tea flavor profile. Specifically, these processes influenced the levels of key compounds such as theanine, glutamic acid, and various carotenoids and flavonoids, which directly impacted taste. Additionally, the modeling of taste receptor complexes, such as the T1R1-EAPKPLV umami complex and the TAS2R39-LALL bitter complex, revealed detailed binding interactions, expanding our understanding of how these compounds contribute to tea flavor. These findings can provide guidance for the optimization processing strategies according to different cultivations, and finally enhance desirable taste attributes and improve the quality of specialized tea products.

## CRediT authorship contribution statement

**Yan Kangni:** Writing – original draft, Investigation. **Yang Jiaqi:** Writing – original draft, Methodology, Investigation. **Zhou Mengxue:** Visualization, Supervision, Methodology. **Peng Qunhua:** Resources, Investigation. **A. Bassiony:** Visualization, Supervision, Methodology. **Bai Xue:** Validation, Methodology. **Feng Shan:** Visualization, Validation, Methodology. **Wang Jiatong:** Visualization, Supervision, Methodology. **Lin Zhi:** Writing – review & editing, Visualization. **Mu Dan:** Writing – review & editing, Visualization, Validation. **Fu Jianyu:** Visualization, Supervision, Methodology. **Wu Yan:** Writing – review & editing, Supervision, Conceptualization. **Lv Haipeng:** Writing – review & editing, Visualization, Validation. **Shi Jiang:** Writing – review & editing, Project administration, Funding acquisition, Conceptualization.

## Ethics statement

The work described in the study carried out in accordance with the World Medical Association Declaration of Helsinki: Ethical principles for medical research involving human subjects. The applied tea samples were safe for consumption. The panelists involved in the sensory evaluation provided informed consent by affirming the following statement before participating in the survey: “I have carefully read the information and fully understand my rights· as a· potential subject in a research experiment. I am aware that my responses are confidential, and I agree to participate in this study.” They had the option to withdraw from the survey at any time without providing a reason. It is important to note that, in China, ethical permission for sensory evaluation is not required by national laws and there is no formal documentation process available for sensory evaluation.

## Declaration of competing interest

The authors declare that they have no known competing financial interests or personal relationships that could have appeared to influence the work reported in this paper.

## Data Availability

No data was used for the research described in the article.

## Appendix A Supplementary data

Supplementary material 1 **Table S1** Characterized taste flavor sensory evaluation. Supplementary material 1

Supplementary material 2 **Table S2** Identified water-soluble peptides in ‘Baiye 1’ collected from low and high altitudes. Supplementary material 2

Supplementary material 3 **Table S3** Predicted results of peptides with potential flavour contribution and bioactivity in ‘Baiye 1’ collected from low and high altitudes. Supplementary material 3

Supplementary material 4 **Table S4** Concentration of 19 essential amino acids and L-theanine in ‘Baiye 1’ collected from low and high altitudes. Supplementary material 4

Supplementary material 5 **Table S5** Concentration of carotenoids in ‘Baiye 1’ collected from low and high altitudes. Supplementary material 5

Supplementary material 6 **Table S6** Concentration of flava-3-ols and flavonoid glycosides in ‘Baiye 1’ collected from low and high altitudes. Supplementary material 6

## References

[bb0005] Ahmed S., Griffin T.S., Kraner D., Schaffner M.K., Sharma D., Hazel M., Cash S.B. (2019). Environmental factors variably impact tea secondary metabolites in the context of climate change. Frontiers in Plant Science.

[bb0010] Bassiony A., Peng Q.H., Baldermann S., Feng S., Yang K.N., Zhang Y.C., Shi J. (2024). Differential accumulation patterns of flavor compounds in Longjing 43 and Qunti fresh leaves and during processing responding to altitude changes. Food Research International.

[bb0015] Chen Y., Han Y., Tong H. (2024). Amino acids and flavonoids analysis reveals quality constituents difference among different albino tea resources. Food Chemistry.

[bb0020] Cheng S., Fu X., Liao Y., Xu X., Zeng L., Tang J., Yang Z. (2019). Differential accumulation of specialized metabolite l-theanine in green and albino-induced yellow tea *(Camellia sinensis*) leaves. Food Chemistry.

[bb0025] Davies M.J. (2005). The oxidative environment and protein damage. Biochimica et Biophysica Acta (BBA) - Proteins and Proteomics.

[bb0030] Dong F., Hu J., Shi Y., Liu M., Zhang Q., Ruan J. (2019). Effects of nitrogen supply on flavonol glycoside biosynthesis and accumulation in tea leaves (*Camellia sinensis*). Plant Physiology and Biochemistry.

[bb0035] Du Z., Ding X., Xu Y., Li Y. (2023). UniDL4BioPep: A universal deep learning architecture for binary classification in peptide bioactivity. Briefings in Bioinformatics.

[bb0040] Ebner J., Baum F., Pischetsrieder M. (2016). Identification of sixteen peptides reflecting heat and/or storage induced processes by profiling of commercial milk samples. Journal of Proteomics.

[bb0045] Fu Y., Zhang Y.H., Soladoye O.P., Aluko R.E. (2020). Maillard reaction products derived from food protein-derived peptides: Insights into flavor and bioactivity. Critical Reviews in Food Science and Nutrition.

[bb0050] Han W.Y., Huang J.G., Li X., Li Z.X., Ahammed G.J., Yan P., Stepp J.R. (2017). Altitudinal effects on the quality of green tea in East China: A climate change perspective. European Food Research and Technology.

[bb0055] Kfoury N., Morimoto J., Kern A., Scott E.R., Orians C.M., Ahmed S., Robbat A. (2018). Striking changes in tea metabolites due to elevational effects. Food Chemistry.

[bb0060] Kottur G., Venkatesan S., Kumar R.S.S., Murugesan S. (2011). Impact of genotype, seasons and manufacturing process on the activities of peptidase and lipoxygenase in tea. European Food Research and Technology.

[bb0065] Kumar M., Selvasekaran P., Chidambaram R., Zhang B., Hasan M., Prakash Gupta O., Amarowicz R. (2023). Tea (Camellia sinensis (L.) Kuntze) as an emerging source of protein and bioactive peptides: A narrative review. Food Chemistry.

[bb0070] Li N., Taylor L.S., Mauer L.J. (2011). Degradation kinetics of Catechins in green tea powder: Effects of temperature and relative humidity. Journal of Agricultural and Food Chemistry.

[bb0075] McGrath B.A., Kinsella M., Huppertz T., McSweeney P.L.H., Kelly A.L. (2016). Proteomic characterisation of heat-induced hydrolysis of sodium caseinate. International Dairy Journal.

[bb0080] Ni T.C., Xu S.S., Wei Y.M., Li T.H., Jin G., Deng W.W., Ning J.M. (2021). Understanding the promotion of withering treatment on quality of postharvest tea leaves using UHPLC-orbitrap-MS metabolomics integrated with TMT-based proteomics. LWT - Food Science and Technology.

[bb0085] Pu D., Shan Y., Qiao K., Zhang L., Sun B., Zhang Y. (2023). Development of an effective protocol for evaluating the saltiness intensity enhancement of umami compounds. Journal of Agricultural and Food Chemistry.

[bb0090] Qiao D., Zhu J., Mi X., Xie H., Shu M., Chen M., Wei C. (2023). Effects of withering time of fresh leaves on the formation of flavor quality of Taiping Houkui tea. LWT - Food Science and Technology.

[bb0095] Shan Y., Pu D., Zhang J., Zhang L., Huang Y., Li P., Zhang Y. (2022). Decoding of the saltiness enhancement taste peptides from the yeast extract and molecular docking to the taste receptor T1R1/T1R3. Journal of Agricultural and Food Chemistry.

[bb0100] Shi J., Wu W., Zhang Y., Baldermann S., Peng Q., Wang J., Lin Z. (2023). Comprehensive analysis of carotenoids constituents in purple-coloured leaves and carotenoid-derived aroma differences after processing into green, black, and white tea. LWT - Food Science and Technology.

[bb0105] Shi J., Yang G., You Q., Sun S., Chen R., Lin Z., Lv H. (2023). Updates on the chemistry, processing characteristics, and utilization of tea flavonoids in last two decades (2001−2021). Critical Reviews in Food Science and Nutrition.

[bb0110] Sun Q., Wu F., Wu W., Yu W., Zhang G., Huang X., Luo L. (2024). Identification and quality evaluation of Lushan Yunwu tea from different geographical origins based on metabolomics. Food Research International.

[bb0115] Tian X., Chen S., Zhong Q., Wang J., Chen J., Chen L., Ma J. (2024). Widely targeted metabolomics analysis reveals the effect of cultivation altitude on tea metabolites. Agronomy.

[bb0120] Wang C.M., Nie C.N., Du X., Xu W., Zhang X., Tan X.Q., Li P.W. (2022). Evaluation of sensory and safety quality characteristics of “high mountain tea”. Food Science & Nutrition.

[bb0125] Wang J., Zhang N., Zhao M., Jing T., Jin J., Wu B., Song C. (2020). Carotenoid cleavage dioxygenase 4 catalyzes the formation of carotenoid-derived volatile β-ionone during tea (Camellia sinensis) withering. Journal of Agricultural and Food Chemistry.

[bb0130] Wu Z.J., Ma H.Y., Zhuang J. (2018). iTRAQ-based proteomics monitors the withering dynamics in postharvest leaves of tea plant (*Camellia sinensis*). Molecular Genetics and Genomics.

[bb0135] Yang Y.Z., Li T., Teng R.M., Han M.H., Zhuang J. (2021). Low temperature effects on carotenoids biosynthesis in the leaves of green and albino tea plant (Camellia sinensis (L.) O. Kuntze). Scientia Horticulturae.

[bb0140] Ye J.-H., Ye Y., Yin J.-F., Jin J., Liang Y.-R., Liu R.-Y., Xu Y.-Q. (2022). Bitterness and astringency of tea leaves and products: Formation mechanism and reducing strategies. Trends in Food Science & Technology.

[bb0145] Yu Q., Huang C., Zhu R., Lu D., Liu L., Lai J., Fan F. (2023). Chemometrics-based investigation of non-volatiles/volatiles flavor of tencha (Camellia sinensis cv. Yabukita, Longjing 43 and Baiye 1). Food Research International.

[bb0150] Yu Z., Yang Z. (2020). Understanding different regulatory mechanisms of proteinaceous and non-proteinaceous amino acid formation in tea (Camellia sinensis) provides new insights into the safe and effective alteration of tea flavor and function. Critical Reviews in Food Science and Nutrition.

[bb0155] Yuan H., Luo Z., Ban Z., Reiter R.J., Ma Q., Liang Z., Li L. (2022). Bioactive peptides of plant origin: Distribution, functionality, and evidence of benefits in food and health. Food & Function.

[bb0160] Zeng L., Fu Y.-Q., Gao Y., Wang F., Liang S., Yin J.-F., Xu Y.-Q. (2024). Dynamic changes of key metabolites in Longjing green tea during processing revealed by widely targeted metabolomic profiling and sensory experiments. Food Chemistry.

[bb0165] Zhang H., Chen Y., Guo Y., Xu W., Wang W., Wu S., Huang Y. (2021). Label-free quantification proteomics reveals the active peptides from protein degradation during anaerobic fermentation of tea. LWT - Food Science and Technology.

[bb0170] Zhang L., Cao Q.Q., Granato D., Xu Y.Q., Ho C.T. (2020). Association between chemistry and taste of tea: A review. Trends in Food Science & Technology.

[bb0175] Zhang L., Zhang L., Pérez-Moreno J., Bin L., Zhang F., Yu F. (2024). Novel umami peptides from two Termitomyces mushrooms and molecular docking to the taste receptor T1R1/T1R3. Food Science and Human Wellness.

[bb0180] Zhang T., Hua Y., Zhou C., Xiong Y., Pan D., Liu Z., Dang Y. (2022). Umami peptides screened based on peptidomics and virtual screening from Ruditapes philippinarum and Mactra veneriformis clams. Food Chemistry.

[bb0185] Zhang Y., Yan K., Peng Q., Baldermann S., Zhu Y., Dai W., Shi J. (2024). Comprehensive analysis of pigment alterations and associated flavor development in strip and needle green teas. Food Research International.

[bb0190] Zhang Y., Yan K., Peng Q., Feng S., Zhao Z., Chen L., Shi J. (2024). Insights into major pigment accumulation and (non)enzymatic degradations and conjugations to characterized flavors during intelligent black tea processing. Food Chemistry.

[bb0195] Zhao F., Qian J., Liu H., Wang C., Wang X., Wu W., Lin Y. (2022). Quantification, identification and comparison of oligopeptides on five tea categories with different fermentation degree by Kjeldahl method and ultra-high performance liquid chromatography coupled with quadrupole-orbitrap ultra-high resolution mass spectrometry. Food Chemistry.

