# Supplementary material for: Comprehensive comparison of potential flavor-active peptides, amino acids and pigments accumulation in different altitudes cultivated albino teas
Source: Food Chem X. 2025 Jul 3;29:102722. doi: 10.1016/j.fochx.2025.102722 (PMC12272920; doi:10.1016/j.fochx.2025.102722)
Supplement: Supplementary material 1 — Table S1 Characterized taste flavor sensory evaluation. [file mmc1.docx]

Supplemetal Information

Table S1 Characterized taste flavor sensory evaluation.

| Sample ID | Tasters | Characterized taste flavor | | | |
| --- | --- | --- | --- | --- | --- |
|  |  | Umami | Mellow | Sweet | Bitter |
| L_GT | Taster 1 | 8.1 | 7.8 | 7.9 | 7.8 |
|  | Taster 2 | 8 | 8.2 | 8.1 | 7.8 |
|  | Taster 3 | 8.3 | 7.9 | 7.6 | 7.9 |
|  | Taster 4 | 8.3 | 7.6 | 8 | 7.5 |
|  | Taster 5 | 8.3 | 8 | 7.8 | 8 |
|  | Taster 6 | 7.8 | 7.8 | 8 | 8 |
|  | **Aver.** | **8.1** | **7.9** | **7.9** | **8.3** |
| H_GT | Taster 1 | 9.4 | 9.3 | 7.8 | 7.9 |
|  | Taster 2 | 9.3 | 9.3 | 7.9 | 7.9 |
|  | Taster 3 | 9.5 | 9.1 | 7.6 | 7.8 |
|  | Taster 4 | 9.3 | 9 | 7.8 | 7.7 |
|  | Taster 5 | 9.3 | 9.2 | 7.9 | 8 |
|  | Taster 6 | 9.4 | 9.2 | 8 | 7.9 |
|  | **Aver.** | **9.4** | **9.2** | **7.8** | **7.9** |

Note: In this study, umami, sweet, bitter, and mellow taste payed attention as characterized taste flavor of the two green tea. During sensory evaluation, 10 score was set as highest value, and 6 score was basic standard. Besides, 0.1 points were set as a differential gradient. The results were integrated in Figure 5C.

A


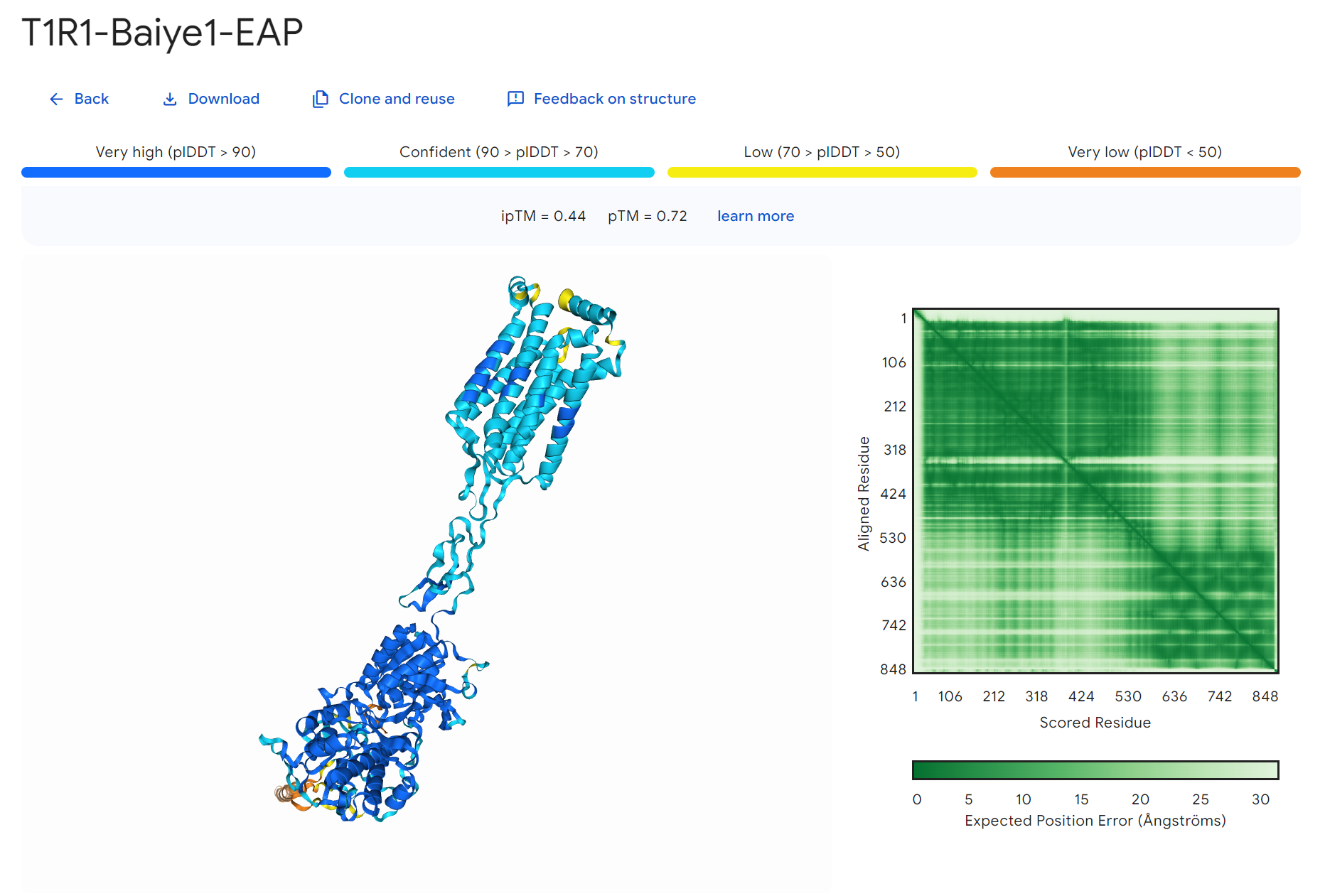

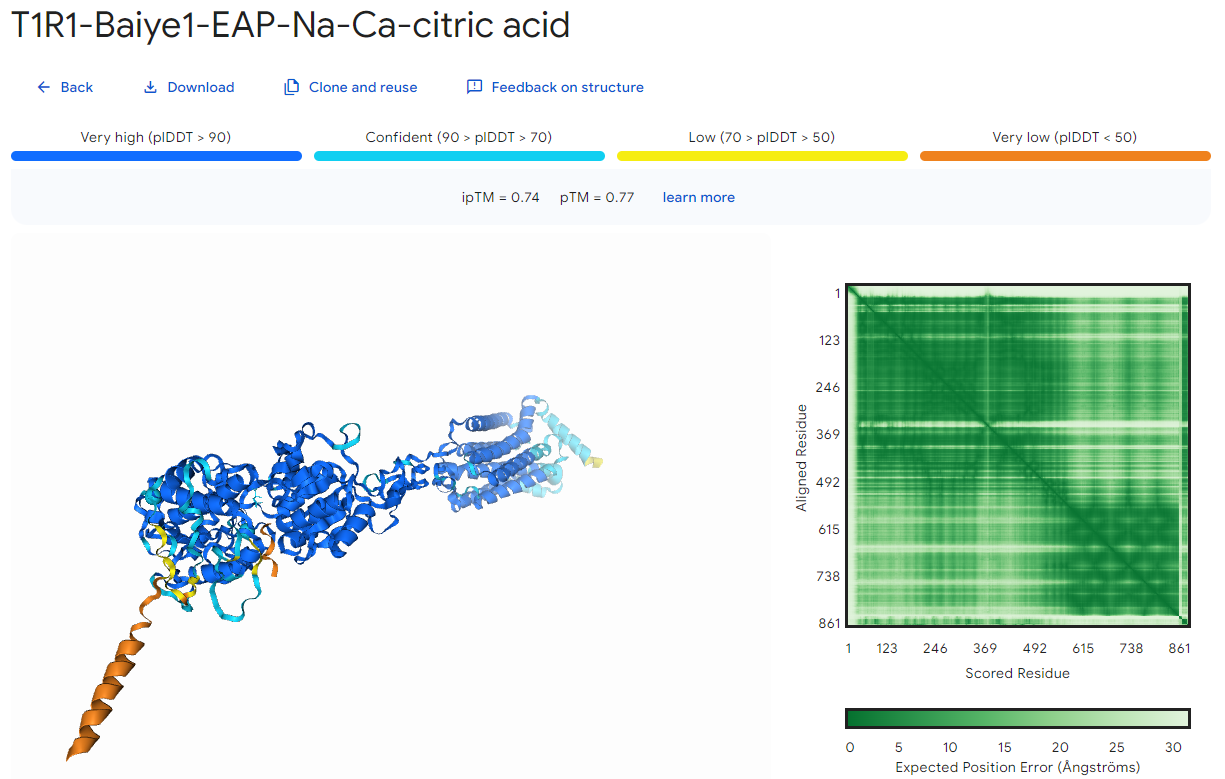


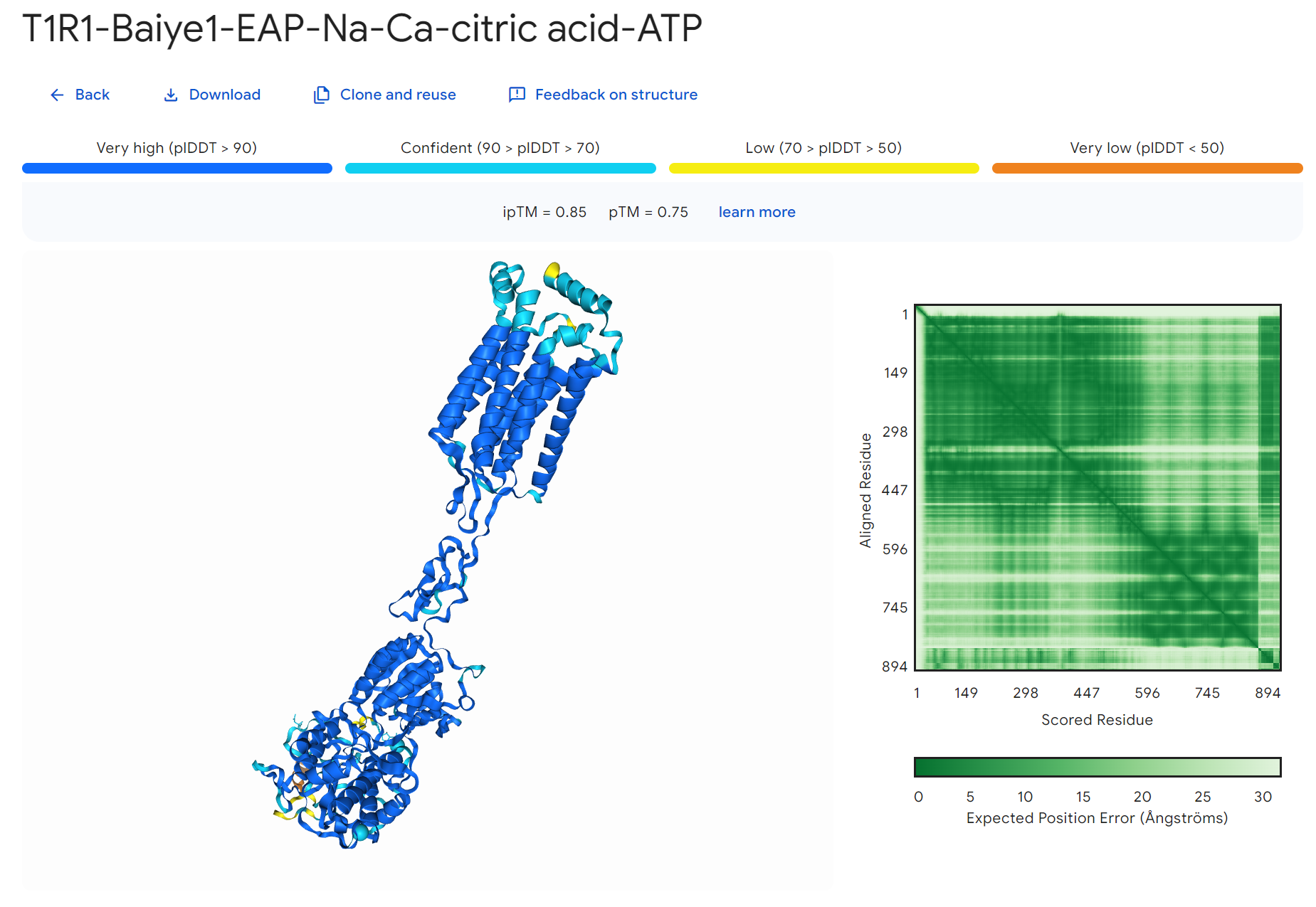


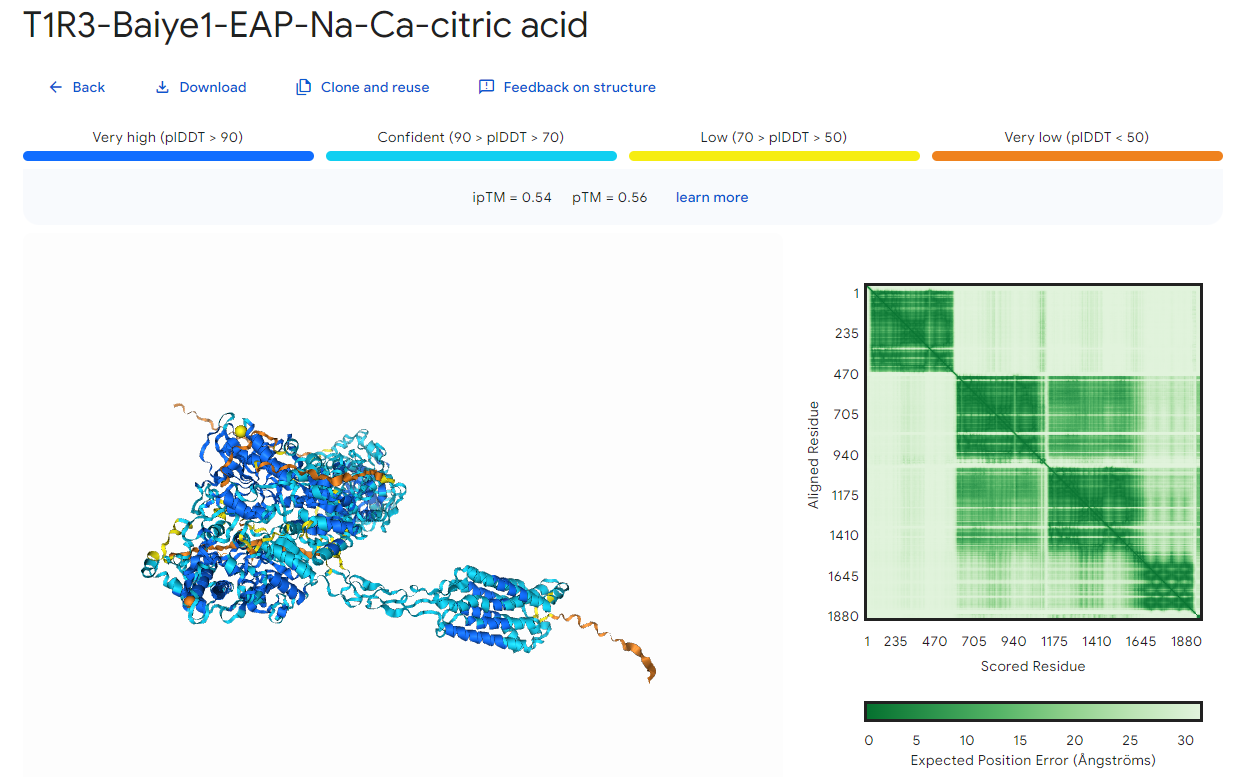


B


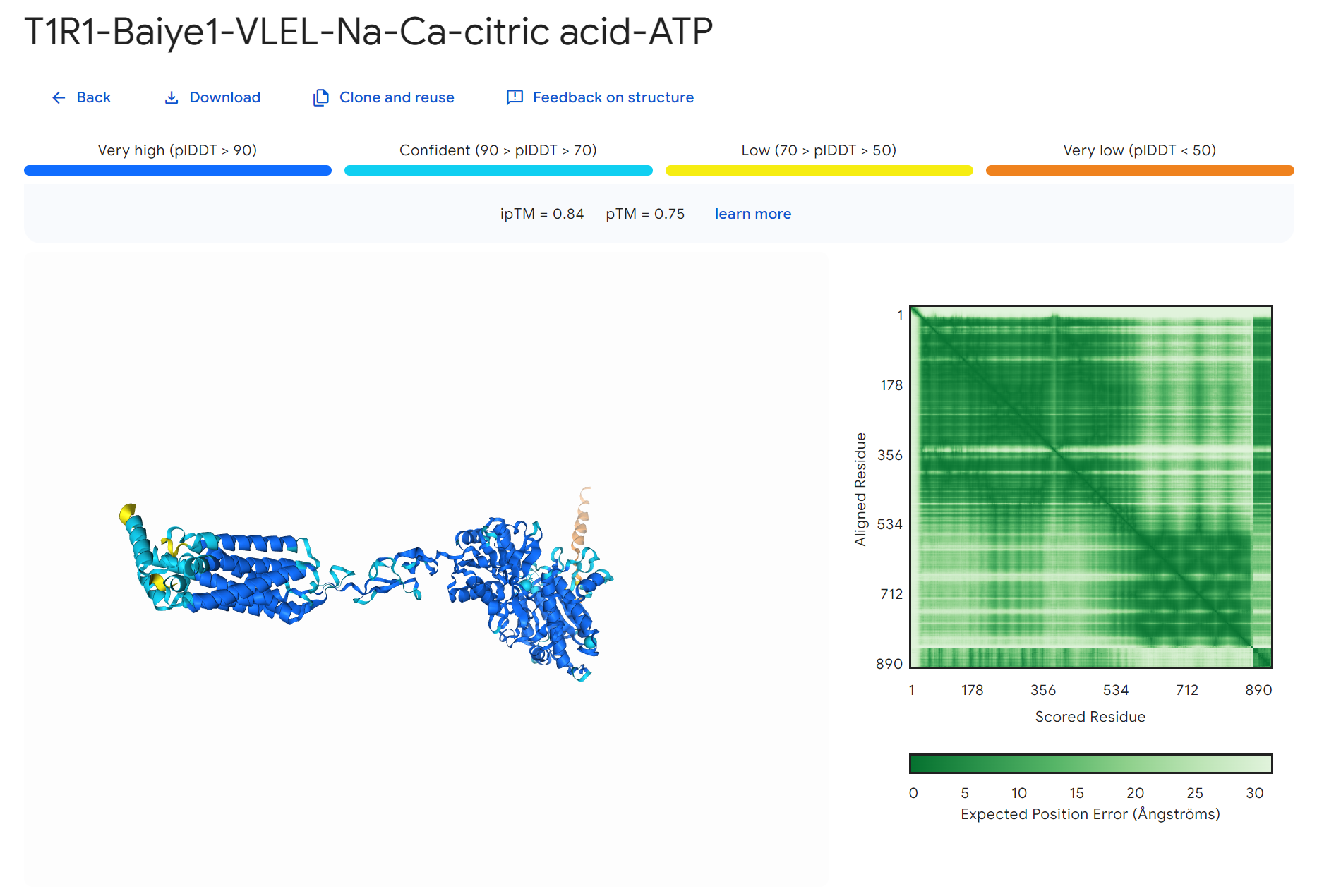


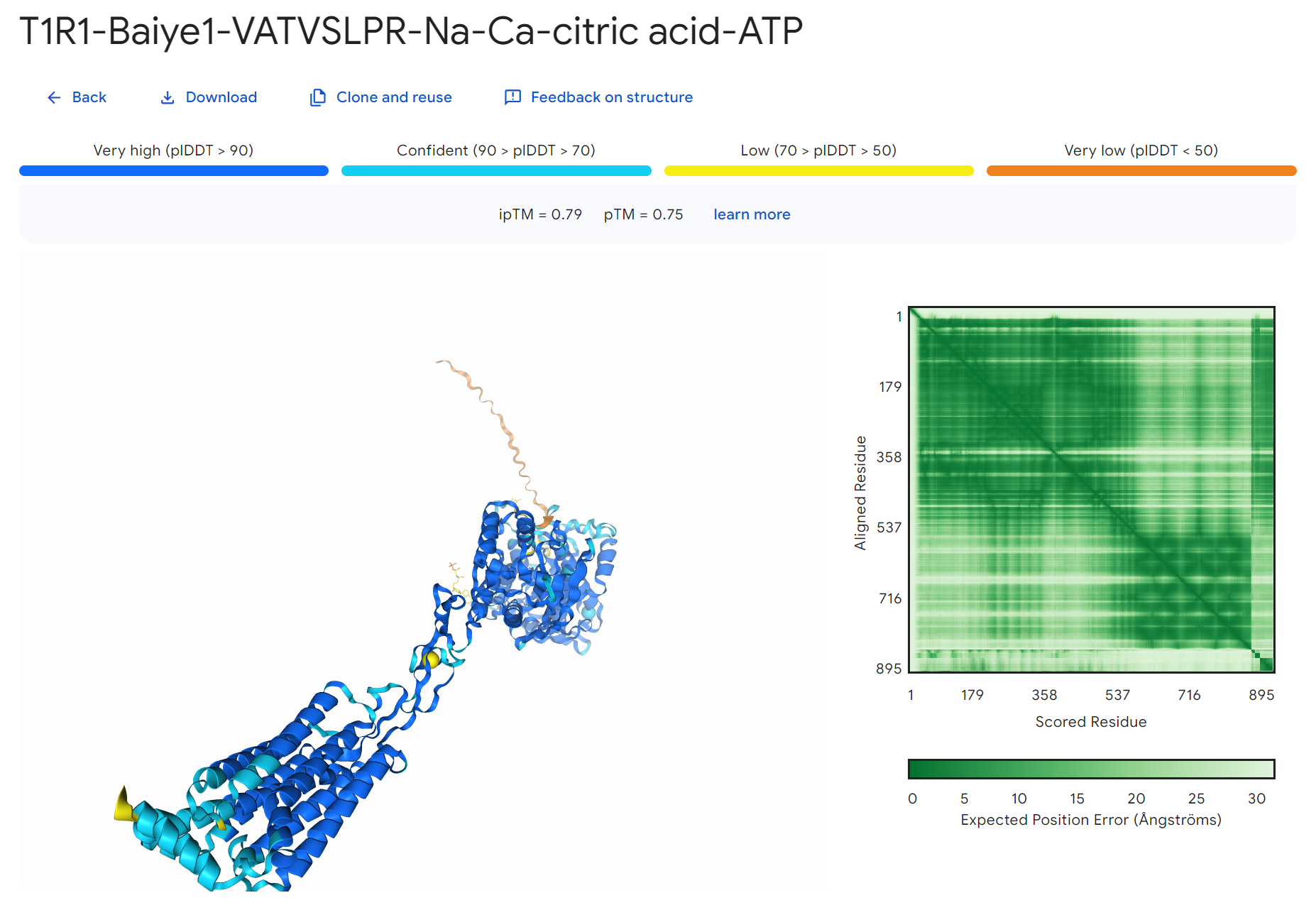


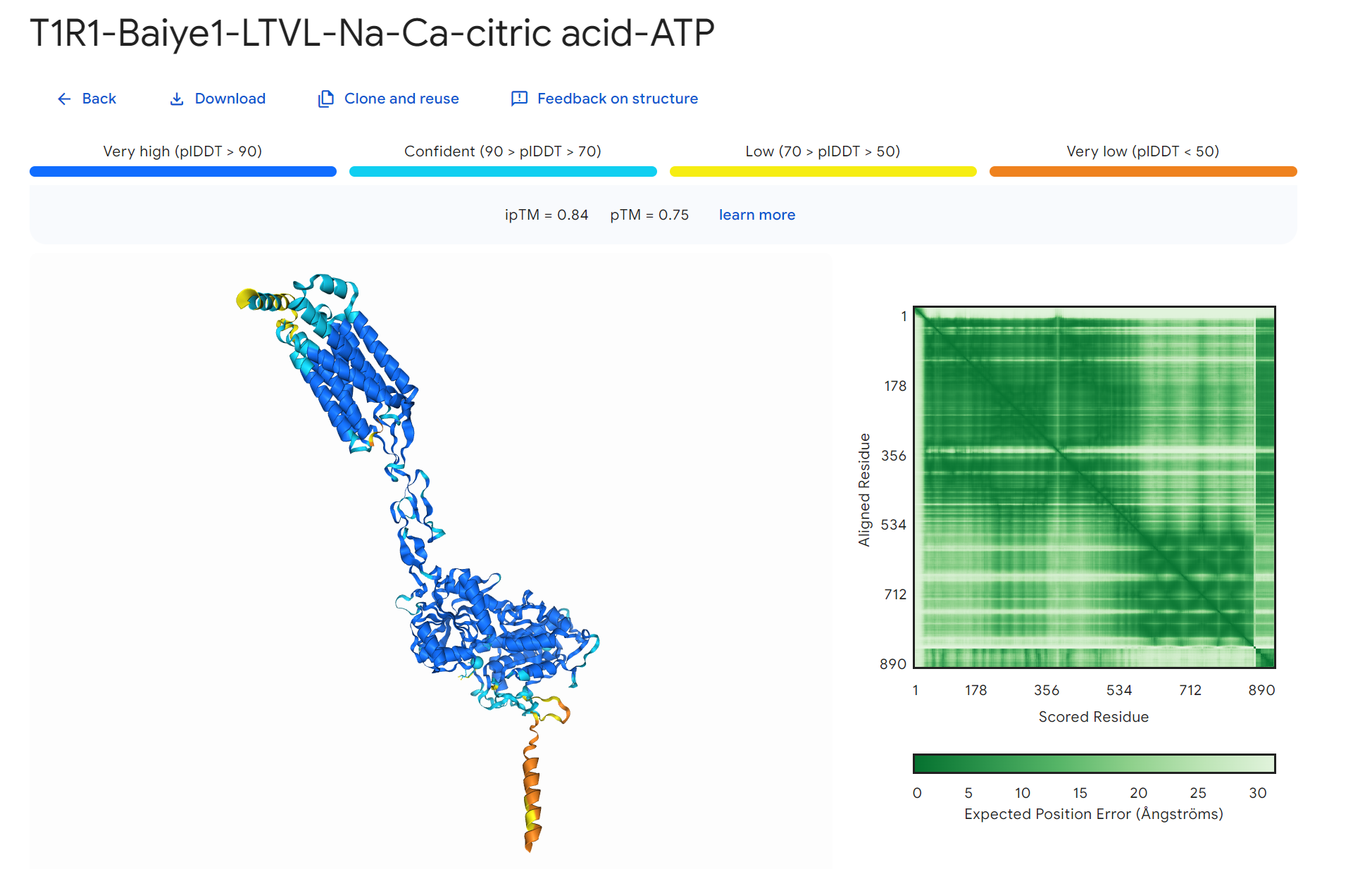


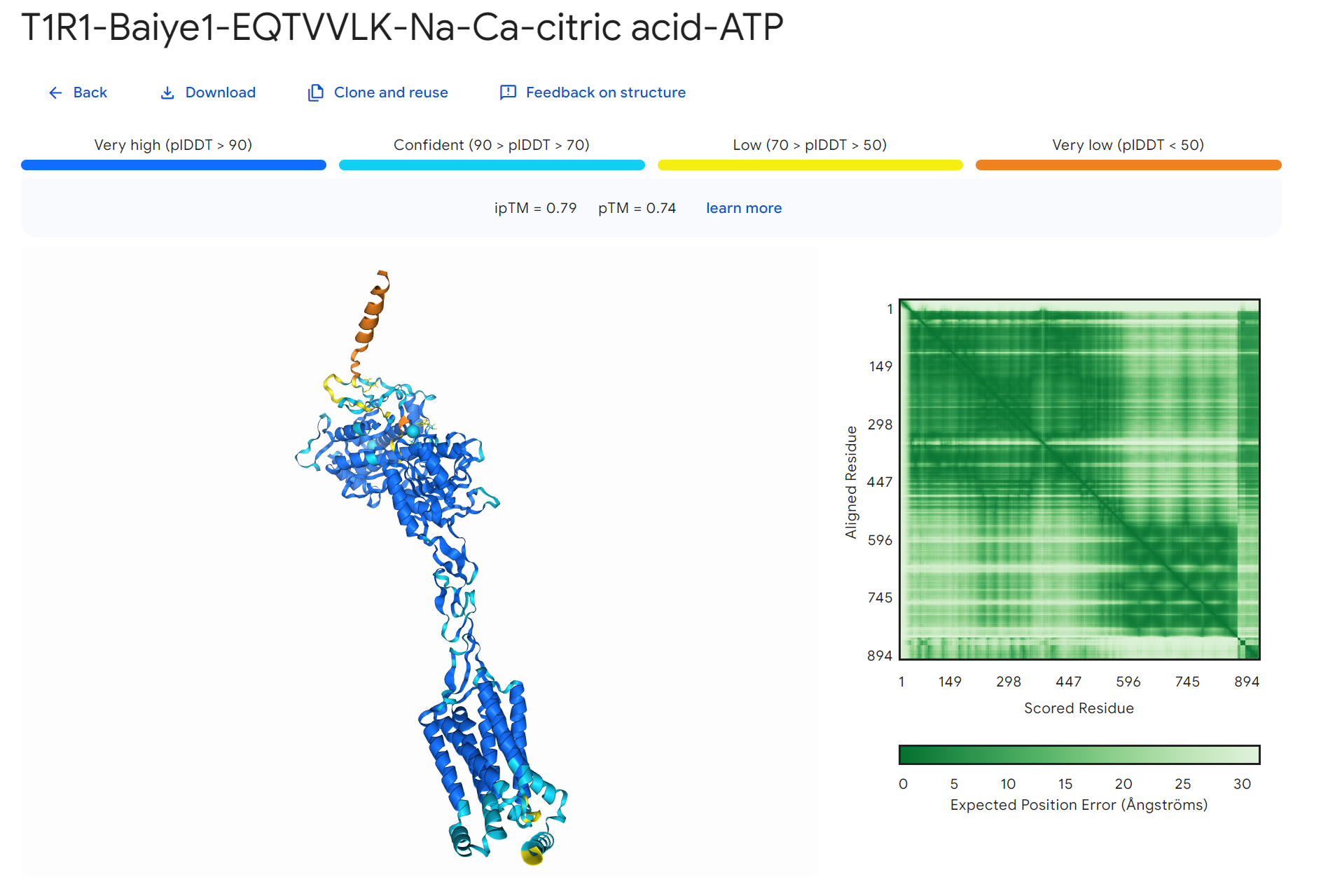


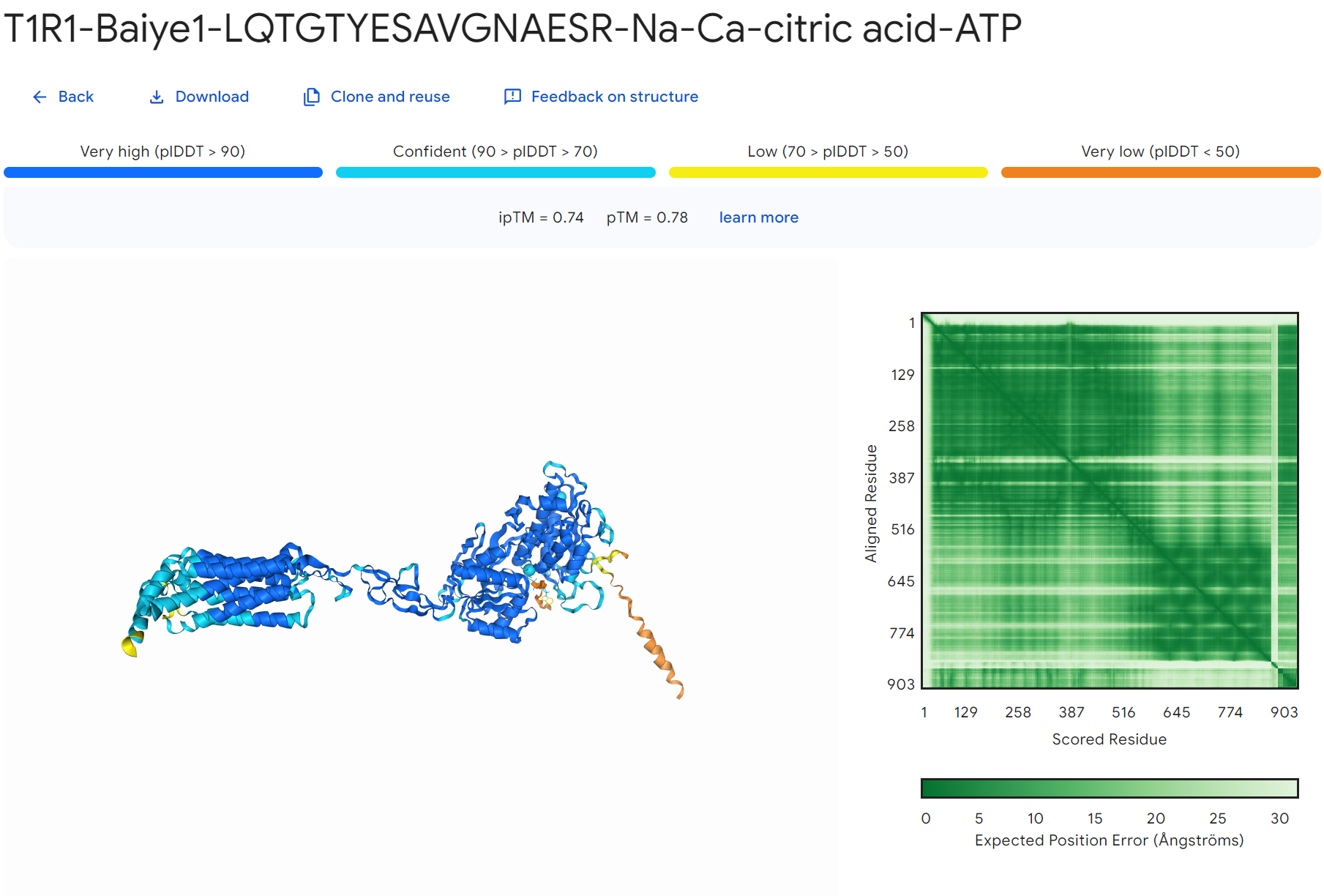


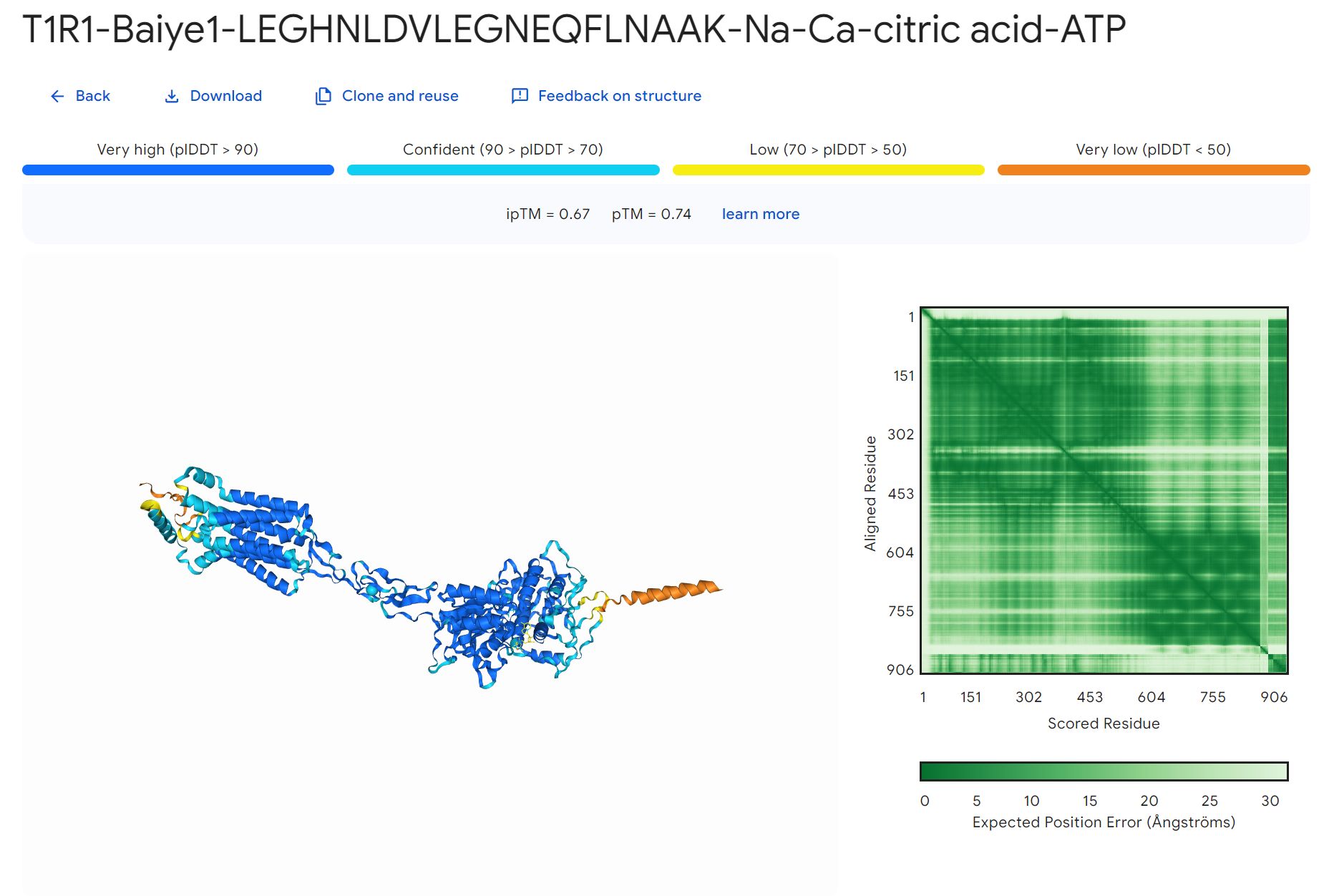


C


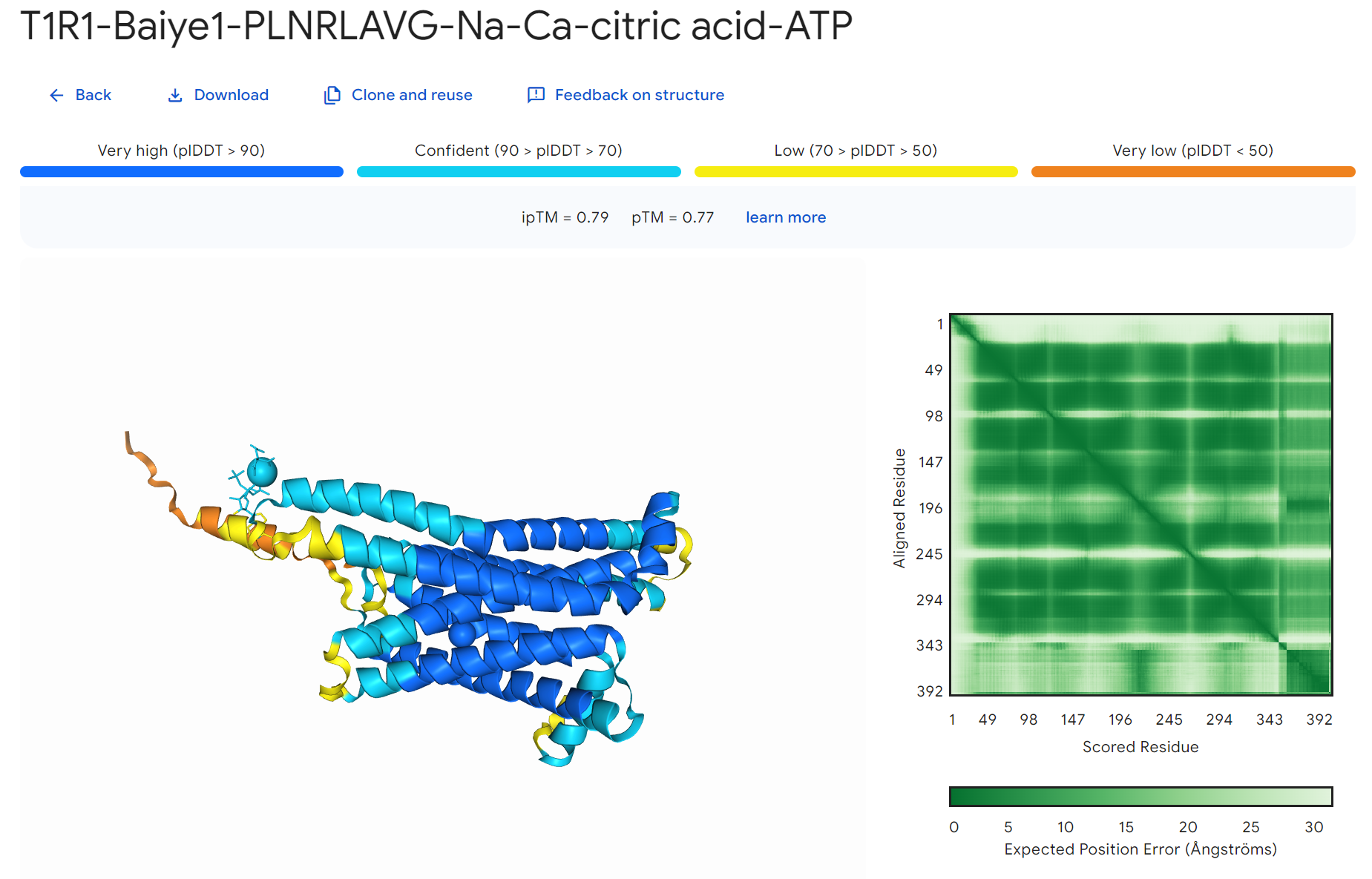


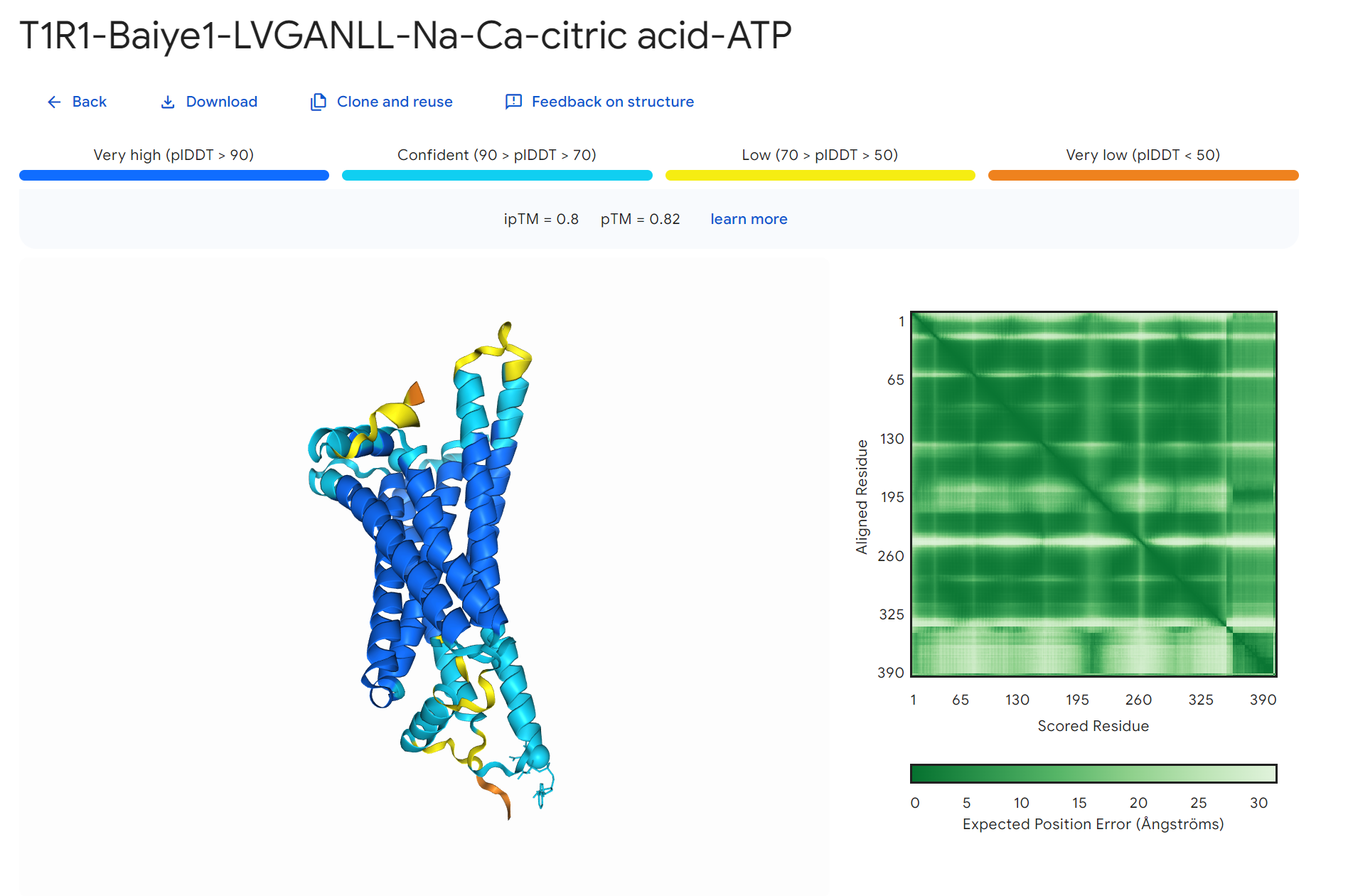


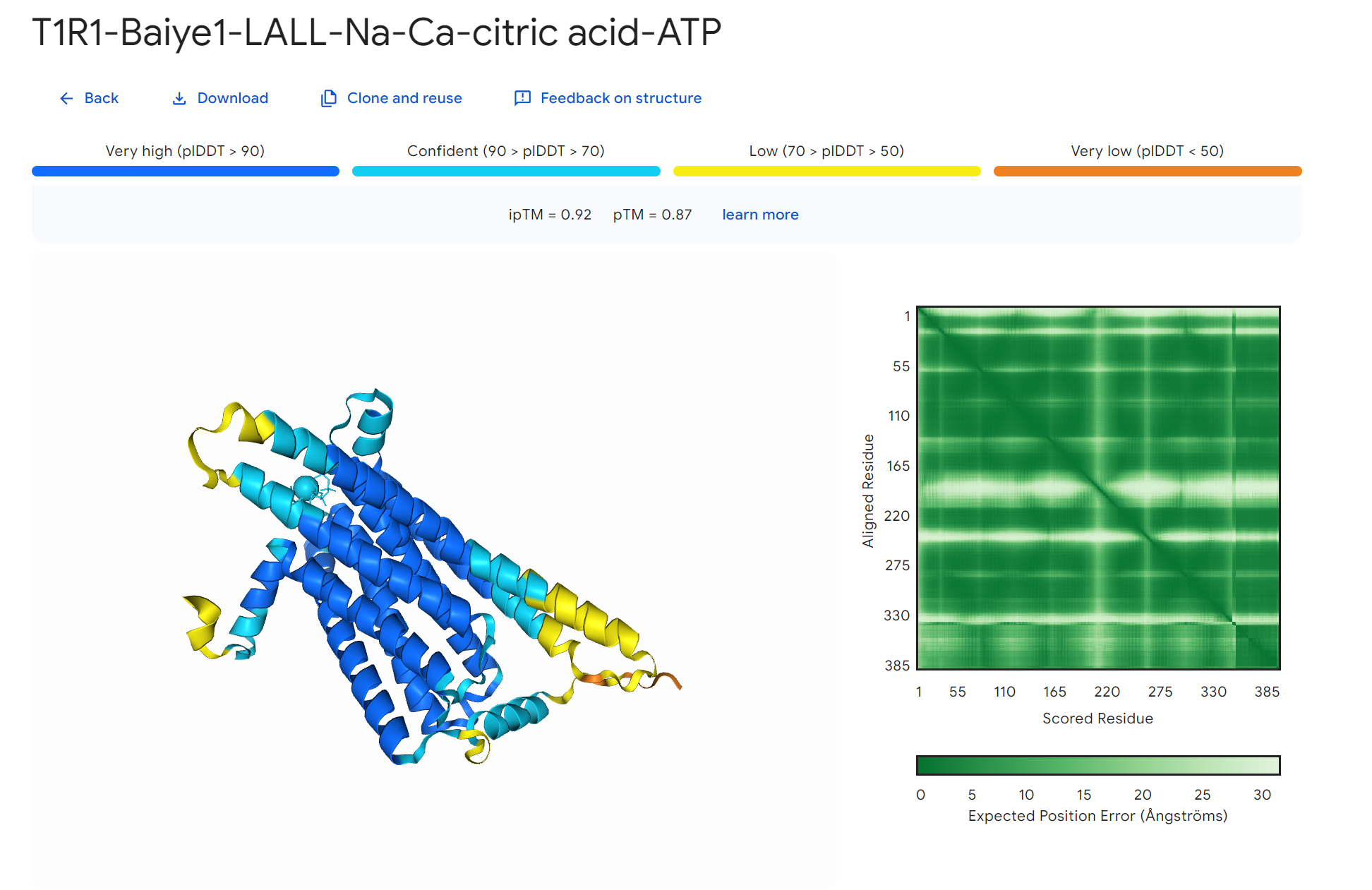


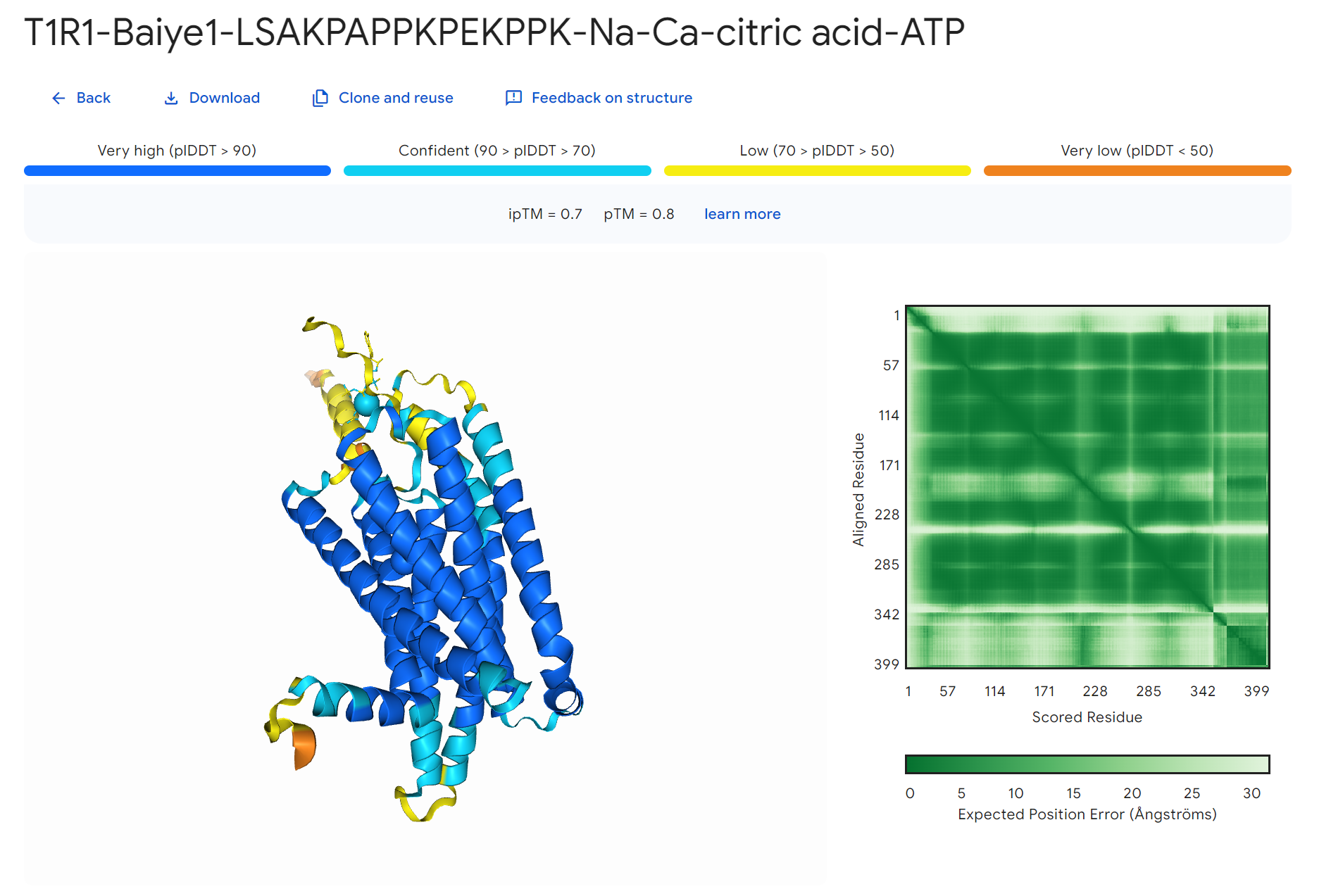


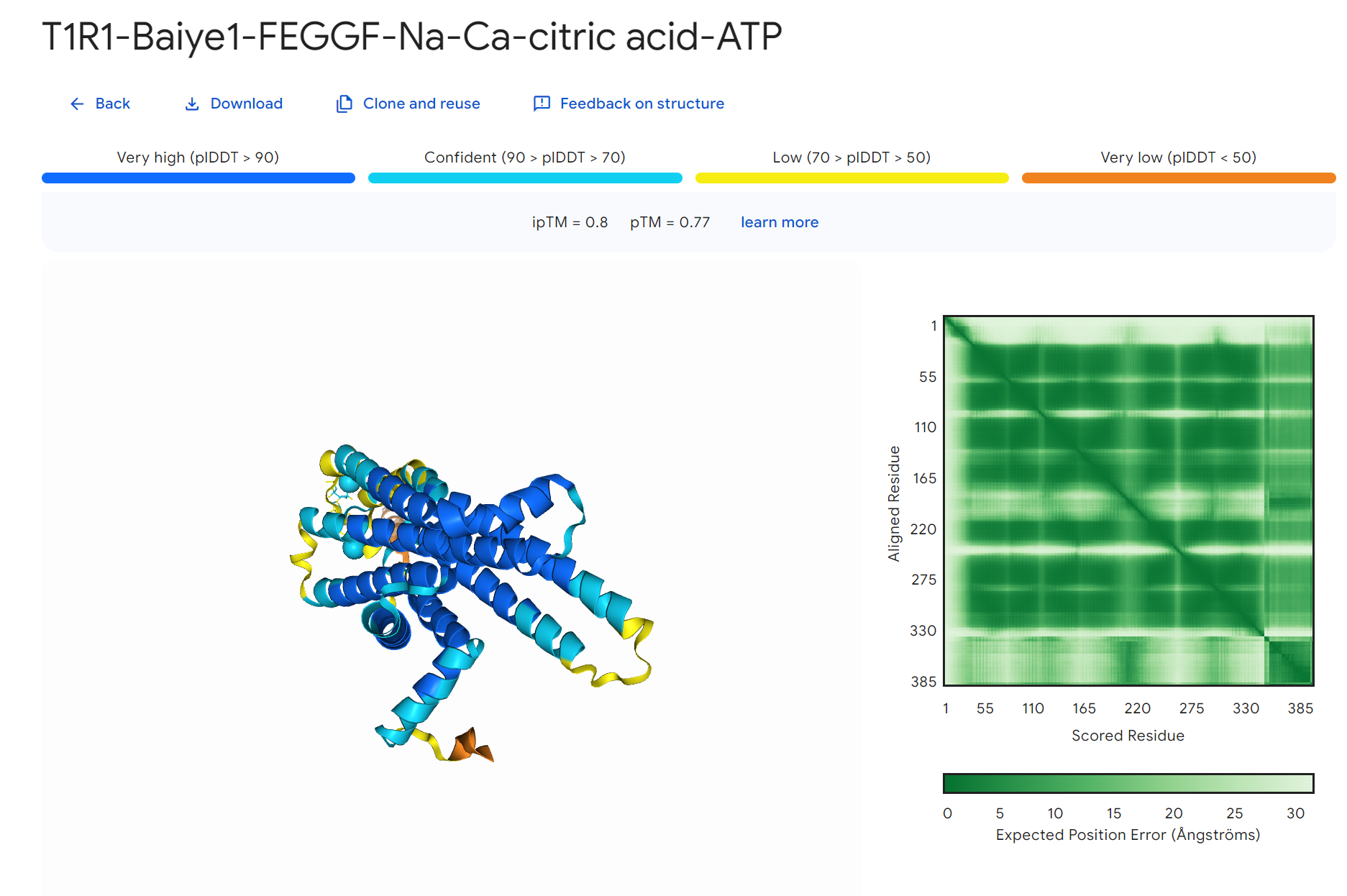


Figure S1
